# Phytochemical profiling and bioactivity validation of *Moringa oleifera* leaves: Antimicrobial, antidiarrheal, analgesic, and in silico insights

**DOI:** 10.1371/journal.pone.0332048

**Published:** 2025-09-12

**Authors:** Mohammad Abdullah Taher, Md. Ashraful Islam, Salsabil Fatima Tasmi, Mohammad Mahmudul Hasan, Hasin Hasnat, Suriya Akter Shompa, Md Rifaet Hossain, Mirola Afroze, Most. Sabila Nazowa, Mala Khan

**Affiliations:** 1 Department of Pharmaceutical Chemistry, Faculty of Pharmacy, Phytochemical Research Laboratory, University of Dhaka, Dhaka, Bangladesh; 2 Bangladesh Reference Institute for Chemical Measurements (BRiCM), Dhaka, Bangladesh; 3 Department of Pharmacy, Pabna University of Science and Technology, Pabna, Bangladesh; 4 Department of Pharmacy, Manarat International University, Dhaka, Bangladesh; 5 Department of Chemistry, Faculty of Science, University of Dhaka, Dhaka, Bangladesh; 6 Department of Pharmacy, School of Pharmaceutical Sciences, State University of Bangladesh, Dhaka, Bangladesh; Universidade Federal do Para, BRAZIL

## Abstract

The increasing threat of antimicrobial resistance and the need for new medicines have renewed interest in medicinal plants like *Moringa oleifera* Lam., a fast-growing tree from the Moringaceae family that can survive in dry conditions. It is easily recognized by its compound leaves and long seed pods. Traditionally, it has been widely used in Ayurvedic medicine and as a nutrient-rich food source, with its leaves, pods, and seeds employed for treating malnutrition, inflammation, and over 300 ailments across South Asia and Africa. A methanolic leaf extract was prepared and analyzed via GC-MS/MS for phytoconstituents. Antimicrobial activity was tested against Gram-positive, Gram-negative bacteria, and fungi using disc diffusion (100 µg/disc), compared to standard antibiotics (30 µg/disc). Antidiarrheal (castor oil-induced model) and analgesic (acetic acid-induced writhing) effects were assessed at 200 and 400 mg/kg doses, with Loperamide and Diclofenac as standards, respectively. Molecular docking analyzed interactions of key compounds with therapeutic targets (URO, EGFR, DHFR, etc.). GC-MS/MS revealed 79 bioactive compounds including 4,5-dimethoxy-2-biphenylcarboxylic acid (14.32%), gamma-sitosterol (3.83%) and stigmasterol (0.81%). The extract showed broad-spectrum antibacterial activity, with strongest inhibition against *Pseudomonas aeruginosa* (19 mm) and *Salmonella typhi* (19 mm), though 30–50% less potent than standard antibiotics. In antidiarrheal testing, 400 mg/kg dose reduced diarrheal episodes by 58.06% compared to control, while in analgesic assay it decreased writhing by 59.18%. Molecular docking demonstrated superior binding of compounds 57, 65 and 61 to molecular targets, with compound 57 showing strongest affinity to URO (−6.7 kcal/mol) and compound 65 to EGFR (−9.6 kcal/mol). ADME/T analysis revealed compounds C57, C59 and C61 possessed optimal drug-likeness (log P 1.33–3.02), high GI absorption, and no predicted toxicity – contrasting with poorly absorbed compounds (C13, C28; TPSA>150). *Moringa oleifera* methanolic leaves extract demonstrated broad-spectrum bioactivity, validating its traditional uses. While less potent than synthetic drugs, its multi-target mechanisms and bioactive diversity highlight its potential as an adjunct therapy. Further studies should isolate active compounds and optimize formulations for clinical applications.

## Introduction

Plants have been a cornerstone of medicinal practices since ancient times, providing therapeutic remedies long before the advent of modern pharmaceuticals [1]. While synthetic drugs dominate contemporary medicine, they often come with severe side effects, making plant-based treatments a safer alternative. Various traditional medicinal systems, including Ayurveda, Unani, and Traditional Chinese Medicine, have long utilized different plant parts for their healing properties [2,3]. According to the World Health Organization, about 80% of people in developing countries still rely on medicinal plants for basic healthcare [4,5]. In developed nations like the United States, nearly one-quarter of prescribed medicines are derived from plants or their chemical components [6]. Medicinal plants are rich in bioactive secondary metabolites with potent antimicrobial, antiviral, anticancer, and cardioprotective properties, offering promising avenues for novel drug development [7]. Over half of the medicines approved by the FDA contain natural compounds or their derivatives, emphasizing the continuing relevance of plants in pharmaceutical advancements [8]. Historical records, including ancient Indian scriptures such as Rigveda and Sushruta Samhita, highlight the longstanding use of medicinal plants, modern research is now confirming many of these traditional claims [9,10]. Unlike synthetic drugs, herbal medicines exhibit fewer adverse effects, making them an attractive alternative for treating pain, oxidative stress, depression, and other serious conditions [11]. The increasing recognition of natural compounds as valuable sources of new, safe, and effective therapeutics underscores the importance of medicinal plants in global healthcare. With ongoing research and a vast reservoir of plant biodiversity, they continue to be a crucial source of innovative treatments, bridging the gap between traditional wisdom and modern medical advancements [6,12].

Plant secondary metabolites, encompassing alkaloids, flavonoids, and steroids, demonstrate potent medicinal activities, serving as vital components in plant defense against herbivores and pathogen (Jain *et al.*, 2019) [13]. These bioactive compounds, extensively utilized in the pharmaceutical industry for their therapeutic properties, contribute not only to the ecological resilience of plants but also provide significant health benefits to humans, presenting valuable resources for drug discovery and development (Jain *et al.*, 2019) [13]. The medicinal importance of secondary metabolites spans over 4000 years, with morphine’s isolation in 1806 marking a transformative era, showcasing the therapeutic potential of these plant-derived compounds [14]. Constituting over 30% of medicinal products, secondary metabolites, including alkaloids and flavonoids, are acknowledged for their diverse structural and therapeutic properties, positioning them as valuable candidates for drug development [15]. Despite historical misconceptions, recent decades have unveiled the significant role of these compounds in pharmaceutical advancements and the treatment of various health conditions, underscoring their enduring importance in medicine [15]. In the context of profiling these molecules from complex mixtures, analytical methods must separate and identify multiple constituents with high specificity.

Gas Chromatography–Mass Spectrometry (GC–MS/MS) combines efficient separation (GC) with precise identification (MS/MS), enabling qualitative and quantitative analysis of volatile/semi-volatile metabolites in plant extracts [16]. This powerful technique allows effective separation and characterization of diverse plant metabolites, making GC-MS pivotal in various research applications [16]. Additionally, Gas Chromatography, particularly with flame ionization and electron capture detectors, is vital in analyzing complex mixtures like essential oils and hydrocarbons, with GC-MS playing an increasingly valuable role in medicinal plant analysis, contributing to herbal drug validation and standardization [17].

Diarrhea remains a major public health concern, especially in low- and middle-income countries, ranking fifth among causes of death in developing nations and eighth globally; in 2019 an estimated 5.7 billion cases led to ~1.1 million deaths [18,19]. While most cases are mild and self-limiting, around 5% of episodes in older children and adults—amounting to nearly 285 million cases annually—progress to moderate or severe illness requiring medical intervention. The primary contributors to diarrheal diseases include poor sanitation, contaminated water, and exposure to pathogenic microorganisms such as bacteria (*Escherichia coli, Salmonella typhi, Shigella flexneri, Staphylococcus aureus*), viruses, parasites, and fungi (*Candida albicans*) [20]. Conventional treatment often relies on antibiotics and antimicrobial agents, but their widespread misuse has led to alarming global challenges, including antimicrobial resistance (AMR), antibiotic-associated diarrhea (AAD), and the emergence of multidrug-resistant (MDR) pathogens [21,22]. Because of this, plant-based antimicrobials are attracting attention as possible alternatives, offering bioactive compounds that may help reduce resistance problems [23,24]. Researchers are increasingly exploring phytochemical-based therapies to address diarrheal diseases while mitigating the drawbacks of conventional antibiotic treatments.

*Moringa oleifera*, also familiar as the “tree of life” or “miracle tree” is a versatile plant recognized for its pharmacological properties and potential benefits against various diseases like ulcers, liver disease, heart disease, and cancer [25,26]. The genus includes 14 species distributed across South Asia, Africa, and the Americas, with *M. oleifera* widely cultivated for both food and medicine [27]. Most of the parts of this plant (root, leaves, fruit, bark, seeds, and flowers) are disclosed to have antitumor, antipyretic, anti-inflammatory, diuretic, cholesterol-lowering, antidiabetic, antioxidant, antispasmodic, antibacterial, and antifungal activities [26,28]. Leaves are nutrient-dense, providing protein (including sulfur-containing amino acids), vitamins (A, B, C), and minerals (calcium, iron), with leaf powder commonly used as a supplement [29]. Alongside, earlier investigations of this genus have reported various bioactive compounds (carotenoids, polyphenol, phenolic acids, flavonoids, alkaloids, glucosinolates, isothiocyanates, tannins, saponins, oxalates, and phytates) were identified in the leaves of *M. oleifera* [30]. Among the bioactive compounds, a previous study reported the highest content of flavonoids (20.76 mg/100 g) and the lowest content of saponins (2.00 mg/100 g). In terms of proximate nutrient composition, carbohydrates were most abundant (46.59%), whereas lipids were present in the lowest amount (7.37%) [31]. Prior docking study on *M. oleifera* leaf extract showed that the phenolic compounds present in the extract had minimum docking scores and strong binding affinity to Human pancreatic alpha-amylase [32].

Accordingly, the present study integrates GC–MS/MS profiling, targeted pharmacological assays (antimicrobial, antidiarrheal, analgesic), and molecular docking/ADME/T analyses to connect chemical composition with observed bioactivity in *M. oleifera*. This integrated approach is intended to generate testable hypotheses for future mechanistic and translational studies.

## Materials and methods

### Plant collection

Leaves of *Moringa oleifera* Lam. were gathered in February 2023 from the campus of Jahangirnagar University, situated approximately 32 km west of the Asian Highway (commonly known as the Dhaka-Aricha corridor). Following standard taxonomic verification procedures, the collected plant material was preserved as a reference specimen at the National Herbarium of Bangladesh (Mirpur, Dhaka) under the catalog number **DACB 93487** for future scientific documentation.

### Drugs and chemicals

All chemicals and reagents used in this study were of analytical grade. Methanol and Tween-80 were obtained from Merck (Darmstadt, Germany). The following pharmaceutical compounds were acquired from commercial sources: diclofenac sodium, azithromycin, amoxicillin, and ciprofloxacin from Beximco Pharmaceuticals Ltd. (Dhaka, Bangladesh), and fluconazole from Square Pharmaceuticals Ltd. (Dhaka, Bangladesh).

### Test microorganisms

For antimicrobial assay, gram-positive bacteria (*Bacillus cereus, Bacillus megaterium, Bacillus subtilis, Staphylococcus aureus, Sarcina lutea*), gram-negative bacteria (*Escherichia coli*, *Pseudomonas aeruginosa, Salmonella paratyphi, Salmonella typhi, Shigella boydii, Shigella dysenteriae, Vibrio mimicus* and *Vibrio parahemolyticus*) and fungi strains (*Aspergillus niger*, *Candida albicans* and *Sacharomyces cerevacae*) were utilized, supplied from State University of Bangladesh.

### Plant material extraction and fractionation

The fresh leaves of *Moringa oleifera* were carefully washed with water and shed-dried for several days until completely dehydrated. The dried plant material was then mechanically ground into a coarse powder using an electric grinder. For extraction, 750 g of the powdered leaves were placed in a 5-liter round-bottom flask and soaked in 2.5 liters of absolute methanol. This mixture was allowed to macerate for 21 days at room temperature with regular manual shaking and stirring to enhance extraction efficiency. After the maceration period, the mixture was sequentially filtered through a cotton plug followed by Whatman No. 1 filter paper to obtain a clear methanolic extract. This extraction process was repeated twice more with fresh methanol to ensure complete extraction of phytoconstituents. The combined filtrates were then concentrated under reduced pressure at 40°C using a Büchi rotary evaporator, yielding 55.89 g of a dark green, viscous methanolic crude extract (MOCME).

### Phytochemical analysis

#### GC-MS/MS analysis methodology.

The phytochemical profiling of leaves of *Moringa oleifera* was performed using gas chromatography-mass spectrometry/ mass spectroscopy (GC-MS/MS) with electron impact ionization. The analysis was carried out on a SHIMADZU GC-MS QP-2020 system equipped with an automated sample handling system consisting of an AOC-20s auto-sampler and AOC-20i auto-injector. Separation was achieved using an SH-Rxi-5MS capillary column (30 m length × 0.25 mm internal diameter; 0.25 μm film thickness) with ultra-pure helium as the carrier gas maintained at a constant flow rate of 1.72 mL/min. The temperature program for chromatographic separation began at an initial temperature of 80°C (held for 2 minutes), followed by a gradual temperature increase of 5°C per minute to 150°C (held for 5 minutes), with a final ramp to 280°C (held for 5 minutes). The injection port temperature was maintained at 220°C, while the ion source temperature was set at 280°C. Samples were introduced in a 5 μL volume with a split ratio of 50:1 using splitless injection mode.

Mass spectrometric detection was performed in electron impact ionization mode at 70 eV, scanning a mass range of 45–350 m/z over a 50-minute acquisition period. The total analysis time was 55 minutes, including a 5-minute solvent delay period. Compound identification was accomplished by comparing acquired mass spectra against reference spectra in the NIST08, NIST08s, and NIST14 mass spectral libraries. The relative abundance of each compound was determined from peak area percentages in the total ion chromatogram. This analytical approach enabled comprehensive characterization of the extract’s chemical composition, including determination of molecular structures and masses of bioactive constituents [33,34].

### *In vitro* analysis

#### Antimicrobial susceptibility testing via disc diffusion method.

The antimicrobial potential of methanol leaves extract of *M. oleifera* was evaluated using the standardized disc diffusion assay [35]. For each bacterial strain, a single Mueller–Hinton agar plate was seeded with standardized inocula. On each plate, five replicate sterile filter paper discs (6 mm diameter) were impregnated with 100 μg of the test sample and placed symmetrically around a central disc containing a standard antibiotic (azithromycin, amoxicillin, or ciprofloxacin, 30 μg/disc). This arrangement allowed direct comparison between the plant extract and the standard within the same plate. Sterile blank discs served as negative controls. Following placement, the plates were refrigerated at 4°C for 24 hours to facilitate compound diffusion, then incubated at 37°C for an additional 24 hours under aerobic conditions. Antimicrobial activity was quantified by measuring the diameter (in millimeters) of the clear inhibition zones surrounding each disc, representing areas of microbial growth suppression [20].

### *In vivo* analysis

#### Animal models and experimental protocol.

For the *in vivo* studies, Swiss albino mice (4–5 weeks old) of both sexes were obtained from the Animal Resources Division at ICDDR,B (International Centre for Diarrhoeal Disease Research, Bangladesh). The animals were housed in polypropylene cages under controlled environmental conditions, including a 12-hour light/dark cycle, ambient temperature of 24 ± 2°C, and relative humidity maintained at 60–70%. Throughout the acclimatization and experimental periods, the mice had free access to standard rodent chow (ICDDR,B formulation) and drinking water.

Prior to experimentation, all animals underwent a 3–4 day acclimatization period to minimize stress-related variables. The study strictly adhered to international guidelines for laboratory animal welfare and received ethical approval from the State University of Bangladesh (SUB), ensuring compliance with international guidelines for humane animal research. The approval was granted under the reference number **2023-U-A4ISUB/A-ERC 1002**, validating adherence to ethical standards in experimental procedures. Following completion of experimental procedures, humane euthanasia was performed using an intraperitoneal injection of ketamine hydrochloride (100 mg/kg) combined with xylazine (7.5 mg/kg) [36].

#### Acute oral toxicity assessment.

Acute toxicity evaluation was conducted according to OECD Guideline 420 (Fixed Dose Procedure). Mice received a single oral administration of *M. oleifera* methanol extract at 2000 mg/kg body weight. Continuous monitoring over 72 hours post-administration revealed no adverse effects, including, no mortality or signs of distress, absence of allergic manifestations, normal behavioral parameters (no sedation or hyperactivity), and maintenance of standard physiological functions Based on these toxicity findings, two safe dosage levels (200 and 400 mg/kg body weight) were selected for subsequent antidiarrheal and analgesic efficacy studies [37].

#### Antidiarrheal activity assessment.

The antidiarrheal potential of methanolic leaves extract of *M. oleifera* was evaluated using the castor oil-induced diarrhea model in Swiss albino mice [38]. The experimental animals were divided into four groups, each containing five mice. The control group received 1% Tween 80 solution in water at a dose of 10 mL/kg body weight, while the positive control group was administered loperamide (5 mg/kg body weight). Two test groups received *M. oleifera* extract at doses of 200 mg/kg and 400 mg/kg body weight respectively. Diarrhea was induced in all animals by oral administration of 1 mL pure castor oil following a 12-hour fasting period with free access to water. Each mouse was housed individually in observation cages with fresh lining paper that was replaced hourly. The number of diarrheal fecal spots was recorded over a 4-hour observation period, with the percentage inhibition of defecation calculated by comparing the test groups to the control group.

The percent inhibition of diarrhea was calculated by following Equation 1.


$$\begin{array}{cc} & \%\;inhibition\;of\;defecation\\ & =\frac{Mean\;number\;of\;defecation\;by\;control-Mean\;number\;of\;defecation\;by\;test\;samples\;or\;standard}{Mean\;number\;of\;defecation\;by\;control}\times 100 \end{array}$$

#### Analgesic activity evaluation.

The analgesic properties of *M. oleifera* leaves extract were investigated using the acetic acid-induced writhing test in mice [34]. The animals were divided into four groups of five mice each. The negative control group received an intraperitoneal injection of 0.1 mL 0.6% acetic acid solution, while the positive control group was pretreated with diclofenac sodium (5 mg/kg body weight) administered orally. Two test groups received oral doses of *M. oleifera* extract at 200 mg/kg and 400 mg/kg body weight respectively. Thirty minutes after treatment, all mice received an intraperitoneal injection of 0.6% acetic acid solution. The characteristic writhing movements, consisting of abdominal constriction followed by hind limb extension, were counted from 5 to 30 minutes post-injection. The percentage inhibition of writhing was determined by comparing the response in test groups to the negative control.

The percentage of writhing inhibition was subsequently calculated using the following formula {2}:


$$\%\;Inhibition\;of\;writhing=\frac{Control\;writhing\;response-Test\;writhing\;response}{Control\;writhing\;response}\times 100$$

### Statistical analysis of results

All experimental data were subjected to statistical analysis using GraphPad Prism version 5.2 software. The results were expressed as mean values with their corresponding standard error of the mean (SEM). Statistical comparisons between groups were performed using one-way analysis of variance (ANOVA) followed by Dunnett’s post-hoc test for multiple comparisons. The threshold for statistical significance was set at probability values of less than 0.05, with more stringent significance levels of less than 0.01 and less than 0.001 also being reported where applicable (*p < 0.5, **p < 0.01, and ***p < 0.001). This analytical approach allowed for rigorous evaluation of the treatment effects while accounting for variability within the experimental groups.

### Molecular docking study

#### Software.

A comprehensive structure-based virtual screening was conducted using *in silico* docking techniques with the AutoDock Vina (version 1.1.2), integrated in PyRx (version 0.9) (http://%28https//pyrx.sourceforge.io/), has performed based on (Table 2) to assess the binding affinities of compounds present in *Moringa oleifera* leaf extract against multiple target proteins [9].

**Table 1:** Selection of target site and grid mapping of target receptors.

Target Activity	Receptor	Standard	Target binding sites	Reference	Grid box
Antioxidant	Urase Oxidase (URO) [PDB ID: 1R4U]	Ascorbic acid (PubChem ID: 54670067)	Gln 31, His 104, Val 29, Trp 106, Arg 105, Thr 28, Thr 107, Cys 103	[46]	Center: x = 26.7241, y = 42.3558, z = 38.3553
					Dimension: x = 61.9199, y = 73.4244, z = 56.0907
	Kelch-like ECH-associated protein 1 (Keap1)[PDB ID: 1X2R]		Leu 365, Leu 557, Ile 416, Arg 415, Ala 366, Ala 510, Gly 417, Gly 364, Gly 603, Ala 366, Val 463, Gly 462, Gly 464, Gly 511, Gly 509, Ala 556	[45]	Center: x = 23.9054, y = 59.0437, z = 7.6944
					Dimension: x = 48.6185, y = 41.2768, z = 59.5298
Cytotoxic	Epidermal Growth Factor Receptor (EGFR)[PDB ID: 1XKK]	Lapatinib (PubChem ID: 208908)	Asp 800, Tyr 998, Leu 1001, Cys 797, Gly 719, Gly 796, Leu 718, Val 726, Lys 745, Asp 855, Thr 854, Phe 997, Glu 804, Tyr 801, Phe 795, Ser 720, Gly 721, Thr 790, Leu 777, Cys 775, Phe 856, Met 766, Leu 788, Leu 758, Leu 844, Asn 842	[47]	Center: x = 19.8531, y = 38.6525, z = 36.5086
					Dimension: x = 53.0174, y = 49.5127, z = 60.8961
	Human epidermal growth factor receptor 2 (HER2)[PDB ID: 1N8Z]		Glu 123, Phe 118, Pro 119, Val 133, Ser 176, Thr 164, Leu 135, Pro 120, Ser 131, Ser 121, Gln 124	[48]	Center: x = 43.6956, y = −56.4719, z = −85.9809
					Dimension: x = 50.3733, y = 73.5836, z = 43.2734
Antimicrobial	Dihydrofolatereductase (DHFR) [PDB ID: 4M6J]	Ciprofloxacin (PubChem ID: 2764)	Ala 9, Val 8, Leu 22, Ile 16, Ser118, Ile 7, Ser 59, Thr56, Gly 117, Lys55, Gly 20, Thr 146, Gly 17, Tyr 121, Trp 24	[49]	Center: x = 11.7243, y = −12.2493, z = −15.3544
					Dimension: x = 43.6316, y = 59.0776, z = 67.0125
	Dihydropteroate synthase(DHPS) [PDB ID: 2VEG]		Leu 208, Thr 209, Asn 213, Phe 239, Arg 238, Gly 205, Lys 237, Lys 210, Asn 242, Ser 147, Ile 150, Phe 151	[50]	Center: x = 35.5558, y = 48.2500, z = 7.1597
					Dimension: x = 50.2681, y = 49.3896, z = 62.5457
Analgesic	Mu Opiod Receptor (MOR) [PDB ID: 5C1M]	Diclofenac (PubChem ID: 3033)	Val 300, Met 151, His 54, Ile 322, Ile 296, Asp 147, Lys 233, Tyr 148, Trp 318, Trp 293, Gly 325, Tyr 326, Gln 124	[40]	Center: x = 2.9902, y = 14.8340, z = −43.9982
					Dimension: x = 68.2525, y = 47.9748, z = 61.3873
	Prostaglandin synthase 2 (PGS2)[PDB ID: 1CX2]		Pro 474, Lys 468, Arg 44, Arg 469, Ser 471, Asn 43, Gln 42, Lys 473, Leu 472, Tyr 122, Thr 70	[52]	Center: x = 25.5184, y = 27.8834, z = 5.1484
					Dimension: x = 66.0163, y = 69.7087, z = 78.2609
Anti-diarrheal	Kappa Opioid Receptor (KOR) [PDB ID: 6VI4]	Loperamide (PubChem ID: 3955)	Tyr 312, Ser 211, Cys 210, Asp 138, Val 108, Leu 135, Thr 111, Tyr 313, Glu 209, Asn 122, Val 207, Ile 208, Ser 123, Val 118, Arg 202, Trp 124, Gln 115, Val 134, Tyr 320	[20]	Center: x = 29.1006, y = −51.0593, z = −22.6624
					Dimension: x = 37.5280, y = 75.7811, z = 57.6389
	Delta-Opioid Receptors (δOR), [PDB ID: 4RWD]		Trp 173, Ile 86, Val 75, Lys 166, Phe 89, Ile 74, Leu 97, Leu 93, Tyr 130, Ser 177, Val 174, Asn 169, Ile 170, Asn 90	[51]	Center: x = −28.1620, y = 7.5599, z = 41.2716
					Dimension: x = 111.8873, y = 68.0193, z = 57.5752

**Table 2:** Identified compounds from methanol extract of leaves of *Moringa oleifera* through GC-MS/MS Analysis along with their reported activites.

Chemical name	Sl No	Compound Name	Retention Time	Area%	m/z	Molecular weight	Formula	Pubchem id	Biological activity	Reference
Alcohol	1	(S)-(+)-2-Amino-3-methyl-1-butanol	4.574	0.1	72	103.16	C_5_H_13_NO	640993	Anti-bacterial and anti-fungal	[55]
	2	1-Dodecanol	10.768	0.1	55	186.33	C_12_H_26_O	8193	Anti-bacterial activity	[56]
	3	D-Allose	11.039	0.87	60	180.16	C_6_H_12_O_6_	439507	Anti-cancer, anti-tumor, anti-inflammatory, anti-oxidative, anti-hypertensive, cryoprotective, and immunosuppressant activities	[57]
	4	cis-Sinapyl alcohol	17.377	0.54	167	210.23	C_11_H_14_O_4_	10130521	Antioxidant, antifungal, antimicrobial, anti-inflammatory, and antinociceptive properties	[58,59]
	5	2-Octylcyclopropene-1-heptanol	20.123	0.56	55	266.5	C_18_H_34_O	534620	No reported activity	–
	6	n-Nonadecanol-1	25.508	1.02	55	284.5	C_19_H_40_O	80281	Anti-inflammatory, antifungal, and antibacterial activity	[60]
	7	Palmitoleamide	23.434	4.1	59	253.42	C_16_H_31_NO	56936054	Antibacterial activity	[61]
	8	1-Eicosanol	28.666	0.37	55	298.5	C_20_H_42_O	12404	Antibacterial activity	[62]
	9	Card-20 [22]-enolide, 3,5,14,19-tetrahydroxy-, (3.beta.,5.beta.)-	30.899	0.14	55	406.5	C_23_H_34_O_6_	258412	No reported activity	–
Aldehydes	10	Tetradecanal	24.684	0.1	55	212.37	C_14_H_28_O	31291	Immunotoxicity activity	[63]
Esters	11	Ethanol, 2-butoxy-	4.667	0.27	57	118.17	C_6_H_14_O_2_	8133	No reported activity	–
	12	Glycerol 1,2-diacetate	8.442	0.43	103	176.17	C_7_H_12_O	66021	No reported activity	–
	13	Methyl-3-methoxy-5-methyl benzoate	11.655	0.08	149	180.20	C_10_H_12_O_3_	13834966	No reported activity	–
	14	Methyl 5-oxo-octadecanoate	9.215	0.07	144	312.5	C_19_H_36_O_3_	3483658	No reported activity	–
	15	Methyl 16-hydroxy-hexadecanoate	26.055	0.08	74	286.4	C_17_H_34_O_3_	3496888	No reported activity	–
	16	1-Docosanol, acetate	27.316	0.15	69	368.6	C_24_H_48_O_2_	69969	Antiviral activity	[64]
	17	1-Methoxy-2-propyl acetate	4.24	1.5	72	132.16	C_6_H_12_O_3_	7946	No reported activity	–
	18	1,2,3-Propanetriol, 1-acetate	6.872	1.28	103	134.13	C_5_H_10_O_4_	33510	No reported activity	–
	19	Carbamic acid, phenyl ester	5.525	0.15	94	137.14	C_7_H_7_NO_2_	69322	No reported activity	–
	20	Methyl 5,9-octadecadienoate (Z,Z)	18.739	0.2	81	294.5	C_19_H_34_O_2_	14150638	No reported activity	–
Ethers	21	Triethylene glycol monododecyl ether	6.975	0.14	57	318.5	C_18_H_38_O_4_	76458	No reported activity	–
	22	Ethanol, 2-(dodecyloxy)-	13.317	0.13	57	230.39	C_14_H_30_O_2_	24750	No reported activity	–
Alkanes	23	Eicosane	13.051	0.07	57	282.5	C_20_H_42_	8222	Antifungal	[65]
Alkenes	24	Squalene	30.381	0.71	69	410.7	C_30_H_50_	638072	Immunity enhancement, skin senility resistance, and hypolipidemic, antioxidant, antitumor, antibacterial, and detoxification effects	[66]
Alkynes	25	1-Decyne	5.99	0.97	67	138.25	C_10_H_18_	12997	Anticholinesterase and antioxidant potentials.	[67]
	26	1-Undecyne	5.87	0.76	67	152.28	C_11_H_20_	75249	No reported activity	–
Phenols	27	Phenol, 2-methoxy-	6.831	0.44	109	124.14	C_7_H_8_O_2_	460	Antioxidant activity	[68]
	28	Phenol, 2,6-dimethoxy-	9.629	0.29	154	154.16	C_8_H_10_O_3_	7041	Antioxidant activity	[69]
	29	Phenol, 2-methoxy-3-(2-propenyl)-	9.695	0.43	164	164.20	C_10_H_12_O_2_	596373	No reported activity	–
	30	Phenol, 3,4-dimethoxy-	10.334	0.17	154	154.16	C_8_H_10_O_3_	16251	Inhibitory mechanism against Xanthomonas oryzaepv. oryzae	[70]
	31	Phenol, 2-methoxy-4-propyl-	10.691	0.07	137	166.22	C_10_H_14_O_2_	17739	No reported activity	–
	32	Phenol, 3,4,5-trimethoxy-	12.021	0.51	169	184.19	C_9_H_12_O_4_	69505	Antioxidant activity	[71]
	33	1,2-Benzenediol, 4-methyl-	8.994	0.42	124	124.14	C_7_H_8_O_2_	9958	No reported activity	–
	34	2-Methoxy-4-vinylphenol	9.285	0.59	150	150.17	C_9_H_10_O_2_	332	Antimicrobial activity	[72]
	35	Benzaldehyde, 4-hydroxy-3,5-dimethoxy-	12.705	0.3	182	182.17	C_9_H_10_O_4_	8655	antioxidant, anti-inflammatory, antimicrobial activities	[73]
	36	(E)-4-(3-Hydroxyprop-1-en-1-yl)-2-methoxyphenol	13.731	1.3	137	180.20	C_10_H_12_O_3_	1549095	Antioxidant activity	[59]
	37	Pinosylvin	23.977	0.45	212	212.24	C_14_H_12_O_2_	5280457	Anti-microbial and antioxidant	[74]
	38	Catechol	7.986	1.58	110	110.11	C_6_H_6_O_2_	289	Anti-bacterial and anti-fungal	[75]
	39	Vanillin	10.167	0.95	151	152.15	C_8_H_8_O_3_	1183	anticancer, antidiabetic, antioxidant, antisickling, antimicrobial, anti-inflammatory, aphrodisiac, cardio-protective, and diuretic actions	[76]
	40	2,6-Dimethoxyhydroquinone	12.595	0.19	127	170.16	C_8_H_10_O_4_	96038	Potent cytotoxic activity against two human cell lines	[77]
	41	Benzeneacetic acid, 4-hydroxy-, methyl ester	25.35	0.18	107	166.17	C_9_H_10_O_3_	518900	No reported activity	–
Sterols	42	Cholest-5-ene, 3-methoxy-, (3.beta.)-	34.964	0.82	165	400.7	C28H48O	79146	No reported activity	–
	43	Campesterol	36.828	0.45	55	400.7	C28H48O	173183	Cholesterol lowering and anticarcinogenic effects	[78]
	44	Stigmasterol	37.307	0.81	55	412.7	C29H48O	5280794	Anticancer, anti-osteoarthritis, anti-inflammatory, anti-diabetic, immunomodulatory, antiparasitic, antifungal, antibacterial, antioxidant, and neuroprotective properties	[79]
	45	gamma.-Sitosterol	38.554	3.83	57	414.7	C29H50O	457801	Antihyperglycemic activity	[80]
Glycols	46	Triethylene glycol monododecyl ether	6.975	0.14	57	318.5	C_18_H_38_O_4_	76458	No reported activity	–
Furans	47	2,4-Dihydroxy-2,5-dimethyl-3(2H)-furan-3-one	5.56	0.11	101	144.12	C_6_H_8_O_4_	538757	No reported activity	–
Ketones	48	2-Propanone, 1-hydroxy-3-(4-hydroxy-3-methoxyphenyl)-	13.562	0.36	137	196.20	C_10_H_12_O_4_	586459	NADPH oxidase inhibitor; anti-inflammatory	[81]
Lactam	49	2-Pyrrolidinone, 1-methyl-	6.291	0.14	99	99.13	C_5_H_9_NO	13387	No reported activity	–
Azole	50	1-β-D-Ribofuranosyl-3-[5-tetraazolyl]-1,2,4-triazole	7.775	0.09	57	269.22	C_8_H_11_N_7_O_4_	545502	No reported activity	–
Nucleosides	51	Guanosine	10.46	0.32	57	283.24	C_10_H_13_N_5_O_5_	135398635	Potent against American Trypanosoma and Leishmania spp	[82]
Carboxylic Acids and Esters	52	Hexanoic acid	5.415	0.16	60	116.16	C_6_H_12_O_2_	8892	Antibacterial Activity	[83]
	53	Nonanoic acid, methyl ester	8.254	0.99	74	172.26	C_10_H_20_O_2_	15606	No reported activity	–
	54	Nonanoic acid	8.708	1.65	60	158.24	C_9_H_18_O_2_	8158	Antibacterial Activity	[84]
	55	8-Nonenoic acid	8.933	0.8	55	154.21	C_9_H_14_O_2_	35698	Inhibits the aquaculture athogen *Saprolegnia parasitica*	[85]
	56	Suberic acid monomethyl ester	10.819	0.12	74	188.22	C_9_H_16_O_4_	554191	Antibacterial Activity	[86]
	57	Pentadecanoic acid, methyl ester	14.702	0.07	74	256.42	C_16_H_32_O_2_	23518	No reported activity	–
	58	Pentadecanoic acid, 14-methyl-, methyl ester	16.192	1.91	74	270.5	C_17_H_34_O_2_	21205	No reported activity	–
	59	Hexanoic acid, 6,6,6-trichloro-, ethyl ester	17.249	0.16	88	247.5	C_8_H_13_Cl_3_O_2_	560519	No reported activity	–
	60	9,11-Octadecadienoic acid, methyl ester, (E,E)-	18.931	0.72	67	294.5	C_19_H_34_O_2_	5365686	No reported activity	–
	61	9-Octadecenoic acid (Z)-, methyl ester	19.035	2.94	55	296.5	C_19_H_36_O_2_	5364509	Anti-inflammatory, antiandrogenic, and anemiagenic properties	[87]
	62	11-Octadecenoic acid, methyl ester	19.122	0.19	55	296.5	C_19_H_36_O_2_	5364432	Anti carcinogenic, anemiagenic, hypocholesterolemic, dermatitigenic	[88]
	63	Hexadecanoic acid, 2-hydroxy-1-(hydroxymethyl)ethyl ester	25.842	0.69	98	330.5	C_19_H_38_O_4_	123409	Antioxidant	[89]
	64	Octadecanoic acid, 2,3-dihydroxypropyl ester	29.035	0.36	57	358.6	C_21_H_42_O_4_	24699	Anticancer, Antimicrobial	[89]
	66	Tetradecanoic acid, 12-methyl-, methyl ester	29.17	0.16	74	256.42	C_16_H_32_O_2_	21206	No reported activity	–
Carbohydrates and Polyols	67	3,4-Altrosan	10.105	0.13	60	162.14	C_6_H_10_O_5_	548229	No reported activity	–
	68	1,2,3,5-Cyclohexanetetrol, (1.alpha.,2.beta.,3.alpha.,5.beta.)-	12.264	0.56	60	148.16	C_6_H_12_O_4_	548226	No reported activity	–
Sugars and Sugar Derivatives	69	D-Mannitol	6.698	0.11	103	182.17	C_6_H_14_O_6_	6251	Used medically as an osmotic diuretic to reduce intracranial and intraocular pressure	[90]
	70	3-O-Methyl-d-glucose	12.439	2.01	87	194.18	C_7_H_14_O_6_	8973	Used as a non-metabolized analogue of glucose by regulating glucose transporter, especially GLUT-4 that regulates the absorption of glucose by insulin into muscle and fat cells	[91]
Biphenyls and Biphenylcarboxylic Acids	71	5-Hydroxy-4-(3-hydroxypropyl)-2-methoxybiphenyl	26.254	1.52	258	258.31	C_16_H_18_O_3_	6425903	No reported activity	–
	72	4,5-Dimethoxy-2-biphenylcarboxylic acid	31.434	35.69	258	258.27	C_15_H_14_O_4_	621031	No reported activity	–
Alcohols and Phenols	73	Resveratrol, trans-	29.543	0.91	228	228.24	C_14_H_12_O_3_	445154	A preventive agent of several important pathologies: vascular diseases, cancers, viral infections, and neurodegenerative processes	[92]
Aromatic Hydrocarbons	74	Azulene	7.855	0.19	128	128.17	C_10_H_8_	9231	Potential compounds in the therapy of dermatological and anticancer diseases	[93]
	75	Naphthalene	8.062	0.88	128	128.17	C_10_H_8_	931	Anticancer, anti-microbial, anti-inflammatory, antiviral, antitubercular, antihypertensive, antidiabetic, anti-neurodegenerative, anti-psychotic, anti-convulsant and anti-depressant activities	[94]
Terpenes	76	5,5,8a-Trimethyl-3,5,6,7,8,8a-hexahydro-2H-chromene	14.434	0.12	124	180.29	C_12_H_20_O	580068	No reported activity	–
	77	Phytol	19.205	0.12	71	296.5	C_20_H_40_O	5280435	Tremendous antinociceptive and antioxidant activities as well as anti-inflammatory and antiallergic effects	[95]
Phosphoric Acid Esters	78	Phosphoric acid, tris(2-ethylhexyl) ester	24.816	0.15	99	434.6	C_24_H_51_O_4_P	6537	No reported activity	–
Phthalates	79	Bis(2-ethylhexyl) phthalate	26.155	0.2	149	390.6	C_24_H_38_O_4_	8343	Antibacterial activity	[96]

#### Ligand retrieval and preparation.

The 3D structures in SDF format of 65 identified compounds out of 79 compounds, along with 5 standard drugs as control: Ascorbic acid (PubChem CID 54670067), Lapatinib (PubChem CID 208908), Ciprofloxacin (PubChem CID 2764), Diclofenac (PubChem CID 3033), and Loperamide (PubChem CID 3955) were retrieved from the PubChem database (https://pubchem.ncbi.nlm.nih.gov/) [33,34,39]. These ligands were imported into the OpenBabel suite within PyRx, where energy minimization was carried out using the default parameters. Following this, the ligands were converted to AutoDock ligand (pdbqt format) for docking using the “Make Ligand” option in PyRx [12,40].

#### Target protein selection and preparation.

To explore the potential activities of *M. oleifera* leaf extract ligands, including their antioxidant, cytotoxic, antimicrobial, analgesic, and anti-diarrheal properties, we targeted two receptors for each activity. The 3D crystal structures of these receptors were obtained from the RCSB protein data bank (PDB) (https://www.rcsb.org/) [41,42]. These structures were then processed by removing co-crystallized ligands and water molecules using the AutoDock 4.2 (MGL Tools 1.5.7) procedure after importing them into Biovia Discovery Studio 4.5. Afterward, we added only polar hydrogens to the cleaned proteins [43]. Finally, Energy minimization was performed with Swiss-PDB Viewer and the structures were subsequently imported into the PyMOL molecular graphics system for visual inspection [44].

For evaluating the antioxidant activity of the compounds, the receptors Urase Oxidase (URO) [PDB ID: 1R4U] [45] and Kelch-like ECH-associated protein 1 (KEAP-1) [PDB ID: 1X2R] [46] were chosen. To measure cytotoxicity, Epidermal Growth Factor Receptor (EGFR) [PDB ID: 1XKK] [47] and Human Epidermal Growth Factor Receptor 2 (HER2) [PDB ID: 1N8Z] [48] were selected. Dihydrofolate reductase (DHFR) [PDB ID: 4M6J] [49] and Dihydroteroate synthase (DHPS) [PDB ID: 2VEG] [50] were chosen for antimicrobial activity. Analgesic activity was assessed using the Mu Opioid Receptor (MOR) [PDB ID: 5C1M] [40] and Cyclooxygenase 2 (COX2) [PDB ID: 1CX2] [33] receptors. Finally, the Kappa Opioid Receptor (KOR) [PDB ID: 6VI4] [20] and Delta Opioid Receptor (δOR) [PDB ID: 4RWD] [51] were selected to assess anti-diarrheal activity.

#### Ligand-protein binding.

Ensuring that ligands bind specifically to their designated macromolecules, the protein was initially configured and optimized (**Table 1**). For antioxidant, cytotoxic, antimicrobial, analgesic, anti-inflammatory, and antidiarrheal activities, the targeted amino acids for each receptor were identified and used for docking simulations as provided in **Table 1**. The final docking was performed using AutoDock Vina (version 1.1.2) to determine the ligands’ affinity for the macromolecules, and the best-fitting 2D and 3D models were predicted using Biovia Discovery Studio (version 4.5) [9].

### ADME/T (Absorption, Distribution, Metabolism, Excretion, and Toxicology) prediction

The selected compounds underwent ADME/T and pharmacokinetics toxicity prediction analysis. Their physicochemical properties, pharmacokinetic behavior, and drug-likeness were assessed using SwissADME (accessed 2025) (http://www.sib.swiss) [34,53], Pre admet (https://preadmet.webservice.bmdrc.https://preadmet.webservice.bmdrc.org/org/) and Admetlab 3.0 (https://admetlab3.scbdd.com/) in accordance with Lipinski’s rules [54]. Additionally, oral toxicity predictions, including Ames test (Salmonella/microsome mutagenicity assay) and hepatotoxicity evaluations, were performed using pkCSM.

## Result

### GC-MS/MS analysis

**Fig 1:**
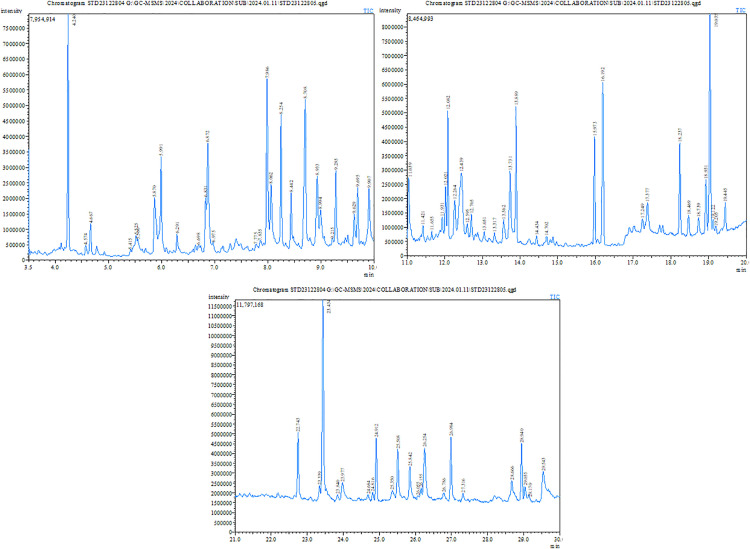
GC-MS/MS chromatogram of methanolic leaves extract of *Moringa oleifera* Lam.

The GC-MS analysis revealed a diverse array of phytochemical constituents present in the extract, with compounds belonging to various chemical classes including alcohols, aldehydes, esters, phenols, sterols, carboxylic acids, and terpenes (**Table 2**, **Fig 1**). The identified compounds showed retention times ranging from 4.24 to 38.554 minutes, with area percentages varying from 0.07% to 35.69%, indicating differences in their relative abundance. Notably, **4,5-dimethoxy-2-biphenylcarboxylic acid** was the most abundant compound (35.69%), followed by **gamma-sitosterol** (3.83%) and **palmitoleamide** (4.1%). Several bioactive compounds with known pharmacological properties were detected, including **squalene** (0.71%), **stigmasterol** (0.81%), **vanillin** (0.95%), **trans-resveratrol** (0.91%), and **phytol** (0.12%).

Compounds with reported biological activities included **antibacterial agents** (1-dodecanol, palmitoleamide, eicosane, catechol), **antioxidants** (phenol derivatives, vanillin, hexadecanoic acid ester), **anti-inflammatory and anticancer agents** (squalene, stigmasterol, trans-resveratrol), and **neuroprotective compounds** (vanillin, phytol). Additionally, some compounds such as **D-allose** (0.87%) and **3-O-methyl-D-glucose** (2.01%) exhibited potential metabolic regulatory effects. However, a significant number of detected compounds had no previously reported biological activities, suggesting the need for further investigation into their pharmacological potential.

### Anti-microbial Activity

The methanolic extract exhibited measurable inhibition against both Gram-positive and Gram-negative bacteria, as well as fungi, though it was generally less potent than standard antibiotics. Among Gram-positive bacteria, *B. cereus* and *S. aureus* were the most susceptible, while *B. subtilis* showed the least sensitivity. In Gram-negative strains, strong inhibition was observed against *P. aeruginosa* and *S. typhi*. Moderate antifungal activity was also recorded against *A. niger* and *C. albicans*. These findings confirm that the extract has broad-spectrum antimicrobial potential, even though activity levels were lower than conventional drugs (**Table 3**).

**Table 3:** The antimicrobial activity of methanol extract of leaves of *M. oleifera* and standard against Gram-positive bacterial and Gram-negative bacteria and fungus.

Test Microorganisms	Azithromycin (30 μg/disc)	Amoxicillin (30 μg/disc)	Ciprofloxacin (30 μg/disc)	*Moringa oleifera* (100 μg/disc)
**Gram Positive Bacteria**				
***Bacillus cereus***	35	33	30	17
***Bacillus megaterium***	33	29	29	13
***Bacillus subtilis***	32	26	33	9
***Staphylococcus aureus***	39	36	34	16
***Sarcina lutea***	37	32	28	14
**Gram Negative Bacteria**				
***Escherichia coli***	39	37	34	18
***Pseudomonas aeruginosa***	42	38	37	19
***Salmonella paratyphi***	32	32	26	16
***Salmonella typhi***	38	31	35	19
***Shigella dysenteriae***	38	30	34	18
***Vibrio mimicus***	33	28	27	13
***Vibrio parahemolyticus***	40	35	34	14
**Fungus**				
***Aspergillus niger***	32	31	33	14
***Candida albicans***	30	34	33	11
***Sacharomyces cerevacae***	33	31	35	10

### Antidiarrheal activity

In the castor oil-induced diarrhea model, *M. oleifera* extract reduced diarrheal episodes in a dose-dependent manner. The higher dose (400 mg/kg) produced a 58.06% reduction compared to the control, approaching the effect of loperamide (77.41%). This indicates that the extract possesses considerable antidiarrheal properties, albeit less pronounced than the standard drug (**Table 4**).

**Table 4:** The antidiarrheal and analgesic effect of methanol extract of *M. oleifera* on castor oil-induced and acetic acid-induced test in mice respectively.

Animal group with respective doses (ml/kg or mg/kg, b.w; p.o)	Antidihreal		Analgesic	
	Number of diarrheal feces (Mean ± SEM)	% reduction of diarrhea	Number of writhing (Mean ± SEM)	% reduction of writhing
**CTL**	10.33 ± 0.33	–	16.33 **± **0.33	–
**STD (Loperamide/Diclofenac sodium)**	2.33 ± 0.33***	77.41	3.67 **± **0.67***	77.55
**MOCME 200**	5.67 ± 0.33***	45.16	10.33 **± **0.67**	36.73
**MOCME 400**	4.33 ± 0.33***	58.06	6.67 **± **0.67**	59.18

Values are expressed as Mean ± SEM (n = 3); CTL = negative control; STD = Positive control; ***p < 0.001, **p < 0.01, *p < 0.05 compared to control compared to negative control.

### Analgesic activity

In the acetic acid-induced writhing test, the extract demonstrated significant analgesic effects. The 400 mg/kg dose reduced writhing responses by 59.18%, compared to 77.55% inhibition by diclofenac. The 200 mg/kg dose also showed moderate activity. These results suggest dose-dependent analgesic potential of the extract (**Table 4**).

### Molecular docking

The data in **Table 5** indicates the binding affinities of different identified compounds from methanolic leaves extract of *M. oleifera* against different receptors. In the redox set, C57 showed the most favorable predicted binding for URO (–6.7 kcal/mol) versus ascorbic acid (–5.3 kcal/mol), while for KEAP1 ascorbic acid scored best (–10.6 kcal/mol) and C57 led the plant constituents (–7.7 kcal/mol). For growth-factor signaling, EGFR was most strongly engaged by C65 (–9.6 kcal/mol), close to lapatinib (–10.0 kcal/mol); C54 and C59 also scored well (–8.3 and –8.2 kcal/mol), and on HER2, C65 matched lapatinib (both –6.9 kcal/mol) with C13, C57, and C61 clustered at –6.6 to –6.7 kcal/mol. In the folate pathway, DHFR docking favored C65 and C61 (–9.1 and –8.3 kcal/mol), comparable to or better than ciprofloxacin (–8.2 kcal/mol), and for DHPS, C61 led (–8.0 kcal/mol) versus ciprofloxacin (–7.2 kcal/mol). For pain/inflammation, MOR docking placed C13 at –7.6 kcal/mol (slightly exceeding diclofenac at –7.3 kcal/mol), with C57, C28, and C59 near –7.1 to –7.3 kcal/mol; COX-2 docking had C28 and C61 at ~–7.7 kcal/mol (at or above diclofenac, –7.0 kcal/mol), with C40 and C56 ~ –7.0 and –6.8 kcal/mol. For GI-motility receptors, KOR docking showed C61 tying loperamide (both –8.9 kcal/mol), with C28, C59, and C54 at –7.9 to –8.0 kcal/mol; δOR docking placed loperamide and C65 highest (both –8.7 kcal/mol), followed by C54 (–8.4 kcal/mol). Collectively, these results nominate a focused subset—particularly C57, C61, C65 (and C54/C59 depending on target)—for experimental follow-up.

**Table 5:** Docking score (kcal/mol) of identified compounds from methanol extract of leaves of *Moringa oleifera.*

Serial	Compounds	Targets									
		URO	KEAP1	EGFR	HER2	DHFR	DHPS	MOR	COX2	KOR	δOR
**C1**	1-Methoxy-2-propyl acetate	−4.0	−4.4	−4.8	−3.5	−4.1	−3.9	−4	−4.3	−4.4	−4.5
**C2**	(S)-(+)-2-Amino-3-methyl-1-butanol	−3.8	−4.4	−4.3	−4.1	−3.7	−3.9	−4.2	−4.7	−4.5	−4.1
**C3**	Ethanol, 2-butoxy-	−3.8	−4	−4.5	−3.6	−4.0	−3.6	−3.8	−4.3	−4.3	−4
**C4**	Hexanoic acid	−4.1	−4.5	−4.8	−4.4	−4.4	−4	−4.2	−5	−4.9	−4.3
**C5**	Carbamic acid, phenyl ester	−5.3	−5.8	−5.9	−4.6	−5.2	−4.8	−5.1	−6.2	−6.2	−5.2
**C6**	2,4-Dihydroxy-2,5-dimethyl-3(2H)-furan-3-one	−5.0	−6	−5.6	−5	−5.2	5.2	−5.2	−5.7	−5.3	−4.9
**C7**	1-Undecyne	−3.9	−4	−5.4	−4.2	−4.3	−4.6	−5.3	−5.5	−5.3	−4.6
**C8**	1-Decyne	−4.0	−3.9	−4.8	−4	−4.4	−4.3	−4.8	−5	−5.1	−4.5
**C9**	2-Pyrrolidinone, 1-methyl-	−3.9	−4	−4.6	−3.5	−3.8	−3.7	−4.1	−4.8	−4.2	−4
**C10**	D-Mannitol	−4.8	−6.2	−5.3	−4.4	−4.5	−4.8	−4.1	--5.5	−5.2	−4.7
**C11**	Phenol, 2-methoxy-	−5.3	−5	−5.4	−4.6	−4.6	−4.4	−5	−5.7	−5.4	−5.4
**C12**	1,2,3-Propanetriol, 1-acetate	−4.1	−5	−5	−4.1	−4.1	−4.4	−4.1	−4.8	−4.3	−4.3
**C13**	1-.beta.-d-Ribofuranosyl-3-[5-tetraazolyl]-1,2,4-triazole	**−7.4**	**−9**	**−7.6**	**−6.7**	**−7.2**	−6.4	**−7.6**	**−8.4**	**−7.7**	−6.7
**C14**	Azulene	−5.8	−5.6	−6.5	−5.4	−6	−5.3	−6	**−7.1**	**−7.1**	−6.1
**C15**	Catechol	−5.2	−5.4	−5.3	−4.6	−4.7	−4.5	−4.7	−5.4	−5.1	−5.4
**C16**	Naphthalene	−5.7	−5.6	−6.6	−5.4	−6.2	−5.2	−5.9	**−7.1**	−6.9	−6.8
**C17**	Nonanoic acid, methyl ester	−4.1	−4.4	−5	−4.2	−4.8	−4.2	−4.7	−4.7	−5.8	−5.1
**C18**	Glycerol 1,2-diacetate	−4.6	−5.3	−5.6	−4.2	−4.8	−4.7	−4.7	−5.6	−5.1	−4.3
**C19**	Nonanoic acid	−4.4	−4.6	−5.3	−4.2	−4.8	−4.2	−4.8	−5.3	−5.5	−5.3
**C20**	8-Nonynoic acid	−4.3	−4.8	**−7.4**	−4.8	−4.7	−4.3	−5	−5.4	−5.4	−4.8
**C21**	1,2-Benzenediol, 4-methyl-	−5.6	−5.9	−5.8	−5.1	−5.1	−5.1	−5.3	−6.1	−5.7	−5.2
**C22**	2-Methoxy-4-vinylphenol	−5.1	−5.6	−5.8	−4.9	−5.2	−4.8	−5.7	−6.4	−6	−6.5
**C23**	Phenol, 2,6-dimethoxy-	−4.8	−5.3	−5.3	−4.7	−4.8	−4.9	−5	−5.6	−5.6	−6.1
**C24**	Phenol, 2-methoxy-3-(2-propenyl)-	−5.5	−5.5	−5.7	−4.6	−5.5	−4.9	−5.6	−5.7	−6.3	−5.4
**C25**	3,4-Altrosan	−5.2	−5.8	−5.3	−4.6	−5.4	−5.1	−5.1	−5.8	−5.4	−4.6
**C26**	Vanillin	−5.3	−5.8	−5.5	−4.4	−5.2	−4.7	−5.2	−6.2	−5.9	−5.2
**C27**	Phenol, 3,4-dimethoxy-	−5.0	−5.5	−5.7	−4.2	−4.8	−4.5	−5	−5.9	−5.7	−5.3
**C28**	Guanosine	**−7.3**	**−10.1**	−6.7	**−6.3**	−6.7	−6.5	**−7.1**	**−7.7**	**−8**	−7.2
**C29**	Phenol, 2-methoxy-4-propyl-	−5.1	−5.8	−5.7	−4.7	−5.4	−5.1	−5.1	−6.9	−6.1	−5.5
**C30**	1-Dodecanol	−4.3	−4.8	−5.3	−4.5	−4.3	−4.4	−4.4	−5.5	−5.5	−5.5
**C31**	Suberic acid monomethyl ester	−4.7	−5.2	−5.5	−4.2	−5	−4.5	−4.9	−5.7	−5.6	−4.5
**C32**	D-Allose	−5.2	−6.7	−5.7	−5.3	−5.4	−5.1	−5.1	−6.2	−5.3	−5.1
**C33**	Methyl-3-methoxy-5-methyl benzoate	−5.6	−6	−5.8	−5.2	−5.9	−5.3	−5.5	−6	−6.2	−6.1
**C34**	Phenol, 3,4,5-trimethoxy-	−5.1	−5.9	−4.9	−4.2	−5	−4.7	−5.2	−5.7	−5.8	−5
**C35**	1,2,3,5-Cyclohexanetetrol, (1.alpha.,2.beta.,3.alpha.,5.bet.)	−5.2	−6.5	−5.7	−4.7	−4.9	−4.6	−4.8	−5.9	−5.1	−4.9
**C36**	3-O-Methyl-d-glucose	−4.8	−6	−5	−4.2	−4.7	−4.7	−5.4	−5.5	−5	−4.5
**C37**	2,6-Dimethoxyhydroquinone	−4.9	−6.2	−5.8	−4.6	−5.1	−4.9	−5.3	−6	−5.7	−6
**C38**	Benzaldehyde, 4-hydroxy-3,5-dimethoxy-	−5.4	−6	−5.4	−4.6	−5.4	−5.2	−5.4	−6.2	−5.6	−5.4
**C39**	Ethanol, 2-(dodecyloxy)-	−4.0	−5.1	−5.7	−4.4	−4.9	−4.5	−5.1	−4.7	−5.9	−5.4
**C40**	2-Propanone, 1-hydroxy-3-(4-hydroxy-3-methoxyphenyl)-	−5.6	−6.6	−6.3	−4.9	−5.8	−5.2	−5.5	**−7**	−6.2	−5.5
**C41**	(E)-4-(3-Hydroxyprop-1-en-1-yl)-2-methoxyphenol	−5.7	−6.5	−6	−5.3	−5.6	−5.1	−5.6	−6.6	−6.3	−6.7
**C42**	5,5,8a-Trimethyl-3,5,6,7,8,8a-hexahydro-2H-chromene	−5.7	−6.7	−5.9	−5.8	−6.1	−5.4	−6.5	−6.1	−7.2	−5.9
**C43**	Pentadecanoic acid, methyl ester	−4.4	−5.3	−5.8	−4.2	−4.8	−4.8	−5.3	−5.4	−6.1	−4.7
**C44**	Pentadecanoic acid, 14-methyl-, methyl ester	−4.0	−5.1	−5.9	−4.6	−5.3	−4.9	−4.7	−6.3	−6.1	−5.5
**C45**	Hexanoic acid, 6,6,6-trichloro-, ethyl ester	−4.5	−5.1	−5.2	−4.2	−5	−4.3	−4.7	−5.4	−6	−4.6
**C46**	cis-Sinapyl alcohol	−5.6	−6.6	−5.9	−4.4	−5.6	−5.8	−6.7	−5.6	−6.1	−4.4
**C47**	Methyl 2-octylcyclopropene-1-heptanoate	−4.7	−5.5	−6.5	−4	−5	−4.4	−4.9	−5.9	−6.9	−5.4
**C48**	Methyl 5,9-octadecadienoate (Z,Z)	−4.8	−5.4	−6.5	−4.2	−5.4	−5.1	−5.6	−5.2	−6.7	−5.7
**C49**	9,11-Octadecadienoic acid, methyl ester, (E,E)-	−4.6	−5.8	−6	−4.1	−5.5	−5.3	−5.4	−6	−6.7	−5.8
**C50**	Phytol	−5.1	−5.9	−6.6	−4.2	−6.2	−5.7	−6.1	−5.9	−6.6	−6.3
**C51**	2-Octylcyclopropene-1-heptanol	−4.9	−5.6	−6.5	−4.7	−5.2	−5.3	−5.6	−5.5	−6.1	−5.6
**C52**	Hexadecanamide	−4.3	−5.4	−6.2	−4.2	−5	−4.8	−5.5	−5.3	−6.3	−5.6
**C53**	Palmitoleamide	−5.0	−5.5	−5.9	−4.3	−5.2	−4.9	−5.6	--5.6	−6.2	−4.9
**C54**	Pinosylvin	**−7.3**	**−8**	**−8.3**	−5.9	**−7.3**	−6.4	**−7.6**	**−8.8**	**−7.9**	**−8.4**
**C55**	Tetradecanal	−4.4	−4.8	−5.6	−4.1	−5.1	−4.7	−4.3	−4.7	−5.5	−4.8
**C56**	Benzeneacetic acid, 4-hydroxy-, methyl ester	−5.4	−5.9	−6.5	−4.6	−5.3	−5	−5.7	−6.8	−7.7	−6.3
**C57**	5-Hydroxy-4-(3-hydroxypropyl)-2-methoxybiphenyl	−6.7	**−7.7**	**−7.4**	**−6.6**	**−7.2**	−5.9	**−7.3**	**−8**	**−7.9**	−6.3
**C58**	Tetradecanoic acid, 12-methyl-, methyl ester	−4.5	−5.4	−6.1	−4.3	−5.1	−4.9	−4.4	−5.1	−6.2	−5.4
**C59**	Resveratrol, trans-	**−7.1**	**−8.3**	**−8.2**	**−6.1**	**−7**	−6.5	**−7.1**	**−8.1**	**−8**	**−7.6**
**C60**	Squalene	−5.8	−6.9	**−7.8**	−4.9	**−7.1**	−5.5	−5.6	**−7.7**	**−8.6**	−5.8
**C61**	Card-20(22)-enolide, 3,5,14,19-tetrahydroxy-, (3.beta.,5.beta.)-	**−7.9**	**−9.5**	**−7.5**	**−6.6**	**−8.3**	**−8**	**−8.4**	**−7.7**	**−8.9**	**−7.7**
**C62**	4,5-Dimethoxy-2-biphenylcarboxylic acid	−6.4	**−7.6**	**−7.3**	−5.8	**−7.3**	−5.9	−6.7	**−7.2**	**−7.8**	−6.1
**C63**	Campesterol	**−7.4**	**−7.1**	**−9.9**	**−7.2**	**−9.4**	**−8.6**	**−9.3**	**−7.5**	**−10.4**	**−9**
**C64**	Stigmasterol	**−7.5**	−5.9	**−9.7**	**−7.4**	**−10**	**−8.8**	**−9.4**	**−7.8**	**−10.4**	−6.1
**C65**	.gamma.-Sitosterol	**−7.4**	**−9.1**	**−9.6**	**−6.9**	**−9.1**	**−8.2**	**−9.3**	**−9**	**−10.5**	**−8.7**
**Standard**	Ascorbic acid	−5.3									
			−6.7								
	Lapatinib			−10.0							
					−6.9						
	Ciprofloxacin					−8.2					
							−7.2				
	Diclofenac							−7.3			
									−7		
	Loperamide									−8.9	
											−8.7

## Discussion

Botanical extracts typically consist of complex mixtures of phytochemicals derived from various natural sources including plants, animals, and microbes. These preparations generally contain between 10−60 different compounds at varying levels of concentration, with their primary biological activity typically attributed to just 2−4 dominant bioactive constituents [97]. The comprehensive analysis of these extracts’ chemical composition can reveal numerous therapeutic properties inherent in medicinal plants. Interestingly, despite the growing interest in phytochemical research, no published studies have yet employed GC-MS/MS techniques to analyze and identify the bioactive compounds present in *M. oleifera*. To bridge this research gap, we conducted a systematic study incorporating detailed GC-MS/MS analysis of this plant species. This GC-MS/MS profiling provided valuable insights into the phytochemical composition of the extract, highlighting the presence of multiple bioactive compounds with potential therapeutic applications. The high abundance of 4,5-dimethoxy-2-biphenylcarboxylic acid, though not extensively studied, may contribute to the extract’s biological properties due to its structural similarity to other biphenyl derivatives reported to exhibit antimicrobial and anti-inflammatory effects [98]. The presence of gamma-sitosterol (3.83%) and stigmasterol (0.81%) is particularly noteworthy, as these phytosterols are well-documented for their cholesterol-lowering, anti-inflammatory, and anticancer properties [79,80]. Based on literature and our profiling, these constituents may help explain traditional uses of the extract in metabolic and inflammatory disorders. Similarly, squalene (0.71%), a triterpene with immunomodulatory and antioxidant effects [66], may contribute to the extract’s potential in enhancing immune responses and preventing oxidative stress-related diseases. Phenolic compounds such as vanillin, catechol, and trans-resveratrol were detected in significant quantities, supporting the extract’s antioxidant and antimicrobial properties [75,76,92]. These findings align with previous studies demonstrating the role of phenolics in scavenging free radicals and inhibiting microbial growth. Additionally, phytol (0.12%), a diterpene alcohol, has been associated with anti-inflammatory and neuroprotective effects [95], suggesting potential applications in neurodegenerative and inflammatory conditions. Interestingly, some detected compounds, such as D-allose and 3-O-methyl-D-glucose, have been linked to antidiabetic and metabolic regulatory effects [57,91] Taken together, and pending bioassay confirmation, these signals may indicate a role in managing diabetes and metabolic syndromes. To clarify scope, any mechanistic statements below are based on molecular docking results and prior literature; they should be considered putative until validated biochemically.

The global emergence of infectious diseases represents an escalating public health crisis, significantly contributing to the development of antimicrobial resistance. This concerning pattern not only threatens population health worldwide but also intensifies the complexities surrounding drug-resistant pathogens [38]. According to World Health Organization projections, antimicrobial resistance may lead to nearly 10 million annual fatalities by 2050 if left unaddressed [99]. Consequently, there is an urgent need to discover novel antibacterial compounds derived from natural sources that can mitigate antimicrobial resistance progression through effective microbial growth suppression. Experimental findings demonstrated considerable antibacterial activity of *M. oleifera* methanolic leaves extract against both Gram-positive and Gram-negative bacterial strains. This antimicrobial activity aligns with existing literature documenting its bioactive potential, which is attributed to secondary metabolites such as flavonoids, tannins, and alkaloids. These compounds are known to interfere with bacterial cell wall synthesis, membrane integrity, and essential enzymatic processes [6]. However, the observed lower efficacy compared to conventional antibiotics suggests that either the active constituents are present in suboptimal concentrations or that bacterial resistance mechanisms reduce their effectiveness. The extract’s relatively stronger inhibition against certain Gram-negative bacteria (*P. aeruginosa*, *S. typhi*) is noteworthy, as Gram-negative species typically exhibit higher resistance to plant-derived antimicrobials due to their outer membrane structure. This finding suggests that certain phytochemicals in Moringa oleifera may interact with outer membrane proteins or lipopolysaccharides (LPS) of Gram-negative bacteria, thereby increasing membrane permeability or weakening efflux pump function. Similar mechanisms have been reported for plant-derived phenolics and sterols, which disrupt bacterial membranes and enhance intracellular drug accumulation [100–102]. These observations warrant further phytochemical and mechanistic investigations.”

The superior performance of synthetic antibiotics underscores their optimized formulations for microbial targeting [103]. However, the growing issue of antibiotic resistance necessitates the exploration of alternative treatments, and plant extracts like *M. oleifera* could serve as complementary or adjunct therapies. Future studies should focus on isolating the specific bioactive compounds responsible for the observed effects, determining minimum inhibitory concentrations (MIC), and evaluating synergistic interactions with existing antibiotics. Additionally, mechanistic inferences should be tested with molecular assays to provide deeper insights into antimicrobial potential. The antidiarrheal results suggest that *M. oleifera* contains bioactive compounds capable of modulating gastrointestinal function. The observed reduction in diarrheal episodes may be mediated—based on literature—by effects on intestinal motility, fluid secretion, or inflammatory pathways. While the extract’s efficacy was somewhat lower than that of Loperamide, the significant reduction at both doses supports traditional use and motivates identification of the responsible phytochemicals.

Acetic acid is well-documented to induce the synthesis of endogenous algogenic substances that activate nociceptive pathways [104]. For this reason, the acetic acid-induced writhing assay serves as a valuable experimental model for evaluating peripheral analgesic activity. This test quantifies pharmacological efficacy by measuring the reduction in characteristic abdominal constrictions – a nocifensive behavioral response resulting from the liberation of natural pain mediators in murine subjects. The analgesic testing revealed that *M. oleifera* extract can attenuate chemically-induced nociceptive responses in a dose-dependent manner (Table 4). The progressive reduction in writhing behavior with increasing doses suggests the presence of multiple pain-modulating compounds in the plant material. The observed effects may result from interactions with various pain pathways, including potential anti-inflammatory activity or direct modulation of nociceptive signaling [33]. While less potent than the standard Diclofenac sodium, the extract’s significant pain-relieving properties at the higher dose indicate its potential as a source of analgesic compounds. These results provide pharmacological validation for traditional uses of *M. oleifera* in pain management and suggest that further characterization of its active constituents could yield valuable therapeutic agents.

**Fig 2:**
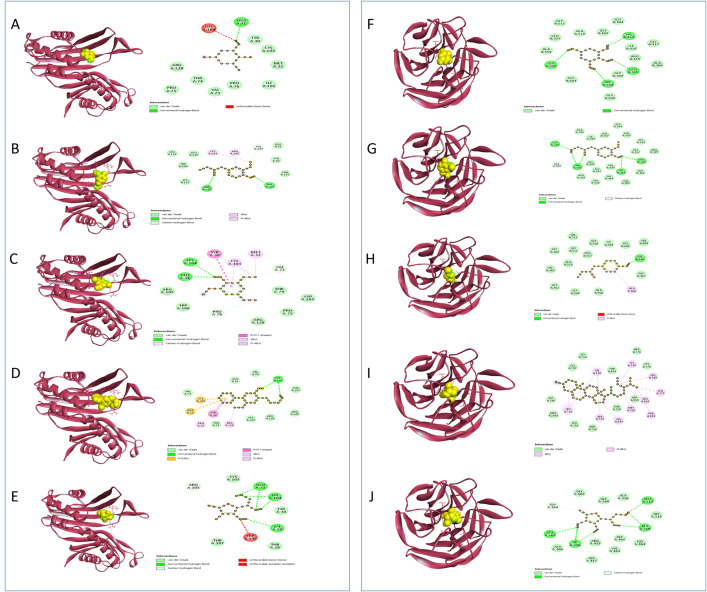
Predicted 3D and 2D interactions of phytocompounds with URO and KEAP1. Panels (A–E) show the binding of C35, C40, C57, C56, and the standard ascorbic acid with URO, respectively. Panels (F–H) depict the binding of C35, C40, C57, C56, and the standard ascorbic acid with KEAP1, respectively.

Uric acid oxidase plays a crucial role in regulating uric acid levels, preventing conditions like hyperuricemia and gout, and indirectly supporting antioxidant defense mechanisms [105,106]. Research shows that the enzyme functions through an exact mechanism including the arrangement and stabilization of response intermediates, with particular steps counting the arrangement and decomposition of urate hydroperoxide into allantoin. By converting uric acid into the more soluble allantoin, Uricase helps to minimize oxidative stress while preserving the beneficial antioxidant properties of uric acid at appropriate levels [107]. Through molecular simulations and experimental tests, it has been appeared that reinforcing hydrogen bonds between subunits can move forward the thermal stability of urate oxidase, hence anticipating its inactivation beneath neutral pH conditions [108]. When association with **URO**, compound 57 builds up one standard hydrogen bond, three pi sulfur bonds, one pi-pi T shaped bond, two alkyl bonds, and one pi alkyl bond, appearing a solid affinity with a binding energy of −6.7 kcal/mol (**Table 6**, **Fig 2**). This is higher than the binding energy of −5.3 kcal/mol watched for ascorbic acid. In differentiate, ascorbic acid connects through three conventional hydrogen bonds, counting one unfavorable donor-donor bond. Compound 56 interatomic with two standard hydrogen bonds, two carbon hydrogen bonds, three alkyl bonds, two pi alkyl bonds, and one pi-pi T shaped bond, coming about in a binding energy of −5.4 kcal/mol.

**Table 6:** Bond and binding site of promising compounds against targeted receptors.

Receptor	Compounds	Binding affinities (kcal/mol)	Bond type	Amino acids
URO	C35	−5.4	Conventional Hydrogen Bond	His A 104
			Unfavorable Donor-Donor	Glu A 31
	C40	−5.6	Conventional Hydrogen Bond	Arg A 128 Trp A 106
			Carbon Hydrogen Bond	His A 104
			Alkyl	Cys A 103
			Pi Alkyl	Arg A 105
	C56	−5.4	Conventional Hydrogen Bond	His A 104, Glu A 31
			Carbon Hydrogen Bond	Pro A 76 Val A 73
			Alkyl	Val A 73 Met A 32 Cys A 103
			Pi Alkyl	Tyr A 30 Cys A 103
			Pi-Pi T shaped	Tyr A 30
	C57	−6.7	Conventional Hydrogen Bond	Trp A 106
			Pi-Sulfur	Met A 32, Cys A 103 Tyr A 30
			Pi-Pi T shaped	Tyr A 30
			Alkyl	Cys A 103 Arg A 128
			Pi Alkyl	Pro A 76
	Ascorbic acid	−5.3	Conventional Hydrogen Bond	Val A 29, His A 104, Glu A 31
			Unfavorable Donor-Donor	Trp A 106
KEAP1	C35	−6.6	Conventional Hydrogen Bond	Val A 604, Val A 463, Gly A 509, Leu A 365
	C57	−7.7	Conventional Hydrogen Bond	Gly A 509 Ile A 416 Ala A 510
			Pi Alkyl	Ala A 366
	C40	−6.7	Conventional Hydrogen Bond	Gly A 367, Val A 606, Val A 463, Ile A 416
			Carbon Hydrogen Bond	Gly A 558
	C56	−5.9	Conventional Hydrogen Bond	Val A 606
			Pi Alkyl	Ala A 366
	Ascorbic acid	−10.6	Conventional Hydrogen Bond	Leu A 365, Ile A 416, Ile A 557, Ala A 510
EGFR	C13	−7.6	Conventional Hydrogen Bond	Asp A 855, Thr A 854
			Pi- Sigma	Thr A 790
			Pi Alkyl	Lys A 745 Met A 766, Leu A 777 Cys A 775
	C54	−8.3	Conventional Hydrogen Bond	Asp A 855, Thr A 854, Lys A 745, Ala A 743
			Pi- Cation	Lys A 745
			Pi- Sigma	Leu A 718
			Pi Alkyl	Val A 726, Leu A 844
	C59	−8.2	Conventional Hydrogen Bond	Leu A 788, Ala A 743
			Carbon Hydrogen Bond	Gly A 796
			Unfavorable Donor-Donor	Thr A 854, Asp A 855
			Pi- Cation	Lys A 745
			Pi- Sigma	Leu A 718
			Pi Alkyl	Val A 726, Leu A 844
	C65	−9.6	Alkyl	Leu A 718, Ala A 743, Val A 726, Met A 766, Leu A 788
			Pi Alkyl	Leu A 844, Phe A 856, Leu A 858, Leu A 777, Lys A 745
	Lapatinib	−10.0	Conventional Hydrogen Bond	Lys A 745, Asp A 855, Thr A 854
			Pi- Anion	Asp A 800
			Pi- Sigma	Val A 729
			Pi-Pi T shaped	Trp A 998
			Pi Alkyl	Leu A 1001, Leu A 718, Cys A 797
HER2	C13	−6.7	Conventional Hydrogen Bond	Thr A 172, Asn A 138
			Pi- anion	Asp A 170
	C28	−6.3	Conventional Hydrogen Bond	Glu A 165, Thr A 172, Ser A 174, Asn A 138
			Pi- anion	Asp A 167
	C57	−6.6	Conventional Hydrogen Bond	Glu A 165, Thr A 164, Ser A 168, Asp A 167
			Carbon Hydrogen Bond	Tyr A 173
			Unfavorable Donor-Donor	Glu A 165
			Pi Alkyl	Pro A 40, Lys A 39
	C61	−6.6	Conventional Hydrogen Bond	Glu A 165, Gln A 166, Ser A 174, Asn A 138
			Carbon Hydrogen Bond	Ser A 168, Asp A 167
	C65	−6.9	Conventional Hydrogen Bond	Glu A 81, Arg A 61
			Alkyl	Pro A 59, Leu A 46, Lys A 45, Val A 58
			Pi Alkyl	Tyr A 55
	Lapatinib	−6.9	Conventional Hydrogen Bond	Pro A 119, Ser A 176
			Carbon Hydrogen Bond	Ser A 176
			Halogen (Fluorine)	Glu A 123
			Pi- Sigma	Val A 133
			Pi-Pi stacked	Phe A 118
DHFR	C13	−7.2	Conventional Hydrogen Bond	Tyr A 121, Thr A 56, Glu A 30
			Pi-Pi T shaped	Tyr A 121
			Carbon Hydrogen Bond	Gly A 117, Ile A 16, Val A 8
			Pi Alkyl	Ile A 16, Val A 8, Ile A 7, Ser A 118
	C54	−7.3	Conventional Hydrogen Bond	Ala A 9
			Pi-Pi stacked	Phe A 34
			Pi-Pi T shaped	Tyr A 121
			Pi Alkyl	Ile A 16, Val A 115
	C57	−7.2	Conventional Hydrogen Bond	Pro A 66
			Unfavorable Donor-Donor	Lys A 68
			Pi- Sigma	Leu A 67
			Pi-Pi stacked	Phe A 34
			Pi-Pi T shaped	Phe A 31
			Alkyl	Pro A 61, Ile A 60
	C61	−8.3	Conventional Hydrogen Bond	Lys A 18, Gly A 20
			Pi- Alkyl	Phe A 34
	C65	−9.1	Pi- Alkyl	Phe A 31, Phe A 34, Ile A 16
	Ciprofloxacin	−8.2	Conventional Hydrogen Bond	Ala A 9
			Carbon Hydrogen Bond	Val A 8, Ser A 118
			Pi Alkyl	Ile A 16, Leu A 22
DHPS	C13	−6.4	Conventional Hydrogen Bond	Leu A 208 Lys A 210 Lys A 237 Arg A 236 Gly A 207
			Pi Alkyl	Lys A 237 Arg A 238
	C28	−6.5	Conventional Hydrogen Bond	Ser A 147 Asn A 213 Phe A 239 Glu A 205
			Unfavorable Donor-Donor	Ser A 147
			Pi Alkyl	Arg A 238
	C57	−6.5	Conventional Hydrogen Bond	Lys A 237, Asp A 201
			Pi- sulfur	Met A 135
			Pi- cation	Arg A 282
			Pi-Pi T shaped	Phe A 206
			Pi Alkyl	Ile A 112
	C61	−8	Conventional Hydrogen Bond	Leu A 208 Ser A 147 Asn A 213
			Alkyl	Arg A 238
	Ciprofloxacin	−7.2	Conventional Hydrogen Bond	Leu A 208 Asn A 231 Phe A 239
			Pi-Donor Hydrogen Bond	Arg A 238
			Alkyl	Arg A 238
			Pi Alkyl	Arg A 238
MOR	C13	−7.6	Conventional Hydrogen Bond	Tyr A 35 Gln A 124 Asn A 127 Asp A 147
			Carbon Hydrogen Bond	His A 54 Ser A 55
			Unfavorable Acceptor- Acceptor	Gln A 124 Ser A 55
			Pi- Sigma	Ile A 322
			Pi-Pi T shaped	His A 319
	C28	−7.1	Conventional Hydrogen Bond	Gln A 124
			Carbon Hydrogen Bond	Gln A 124
			Unfavorable Acceptor- Acceptor	Tyr A 326
	C54	−7.6	Conventional Hydrogen Bond	Lys A 233
			Pi- Sigma	Val A 300 Val A 236
			Pi Alkyl	Ile A 322 Ile A 296
	C57	−7.3	Conventional Hydrogen Bond	Tyr A 148 Ile A 322
			Carbon Hydrogen Bond	His A 54
			Pi Alkyl	Val A 300 Val A 236 Ile A 296 Lys A 233 Leu A 232
	C59	−7.1	Conventional Hydrogen Bond	His A 54 Tyr A 326 Lys A 233
			Pi- Sigma	Val A 300 Val A 236
			Pi Alkyl	Ile A 322
	Diclofenac	−7.3	Halogen (cl, br, I)	Met A 151
			Unfavorable Donor-Donor	Asp A 147
			Pi-Pi stacked	His A 54
			Pi Alkyl	Val A 300 Val A 236 Ile A 322 Ile A 296
COX2	C28	−7.7	Conventional Hydrogen Bond	Cys A 47 Val A 155 Asp A 157
			Pi Alkyl	Ala A 156 Pro A 154
	C40	−7	Conventional Hydrogen Bond	Ala A 202
			Carbon Hydrogen Bond	His A 207
			Pi-Pi T shaped	His A 388
			Pi Alkyl	His A 207 Ala A 202 His A 388 His A 386
	C56	−6.8	Conventional Hydrogen Bond	Tyr A 385 Thr A 206
			Unfavorable Acceptor- Acceptor	Ala A 202
			Pi Alkyl	Ala A 202
			Pi-Pi T shaped	His A 388
	C61	−7.7	Alkyl	Arg A 841 Cys A 797 Leu A 844
	Diclofenac	−7	Conventional Hydrogen Bond	Asn A 43, Ser A 471 Arg A 469
			Amide Pi stacked	Asn A 43 Lys A 468
			Pi- cation	Lys A 468
			Pi Alkyl	Pro A 474 Arg A 44
KOR	C28	−8	Conventional Hydrogen Bond	Val A 155 Asp A 157 Cys A 47
			Pi Alkyl	Ala A 156 Pro A 154
	C54	−7.9	Conventional Hydrogen Bond	Tyr A 385
			Unfavorable Donor-Donor	Trp A 387
			Pi- Sigma	Leu A 391
			Pi-Pi T shaped	His A 388
			Amide Pi stacked	Trp A 387
			Pi Alkyl	Val A 295
	C59	−8	Conventional Hydrogen Bond	Tyr A 385 Trp A 387
			Pi –Donor Hydrogen Bond	Phe A 395
			Pi-Pi T shaped	His A 388
			Amide Pi stacked	Ala A 202
			Pi- Sigma	Leu A 391
			Pi Alkyl	Val A 295
	C61	−8.9	Conventional Hydrogen Bond	Asp A 138 Arg A 138
			Pi-Pi T shaped	His A 388
	Loperamide	−8.9	Carbon Hydrogen Bond	Tyr A 312 Cys A 210
			Pi- anion	Asp A 138
			Pi-Pi stacked	Tyr A 312
			Alkyl	Val A 108
δOR	C54	−8.4	Conventional Hydrogen Bond	Val A 70 Cys A 328
			Carbon Hydrogen Bond	Gly A 73
			Pi-Pi stacked	Phe A 329
			Pi Alkyl	Ala A 319 Pro A 315 Val A 316 Cys A 328 Leu A 69
	C59	−7.6	Conventional Hydrogen Bond	Pro A 315
			Pi-Pi T shaped	Phe A 329
			Amide Pi stacked	Leu A 65
			Pi Alkyl	Leu A 65 Ala A 319 Leu A 69 Val A 62 Val A 70
	C61	−7.7	Conventional Hydrogen Bond	Ser A 42 Ser A 45
			Carbon Hydrogen Bond	Glu A 112
			Unfavorable Acceptor- Acceptor	Ser A 44
	C65	−8.7	Carbon Hydrogen Bond	Val A 75
			Pi- Sigma	Trp A 173 Phe A 89
			Pi Alkyl	Trp A 173 Phe A 89
			Alkyl	Leu A 93 Lys A 166 Ile A 86 Val A 75
	Loperamide	−8.7	Pi-Pi stacked	Trp A 173 Phe A 89
			Pi Alkyl	Phe A 89 Lys A 166 Ile A 86
			Alkyl	Ile A 86 Ile A 74 Val A 75

Under normal circumstances, **KEAP1** interacts with Nrf2 in the cytoplasm and encourages its degradation. During oxidative stress, alterations in **KEAP1**’s thiol groups lead to the release of Nrf2, which at that point moves to the nucleus and enacts the transcription of various cytoprotective genes by binding to the antioxidant response element (ARE) in the DNA. The KEAP1-NRF2 pathway is linked to several diseases associated with oxidative stress, including cancer, neurodegenerative disorders, and inflammatory conditions. Therapeutic procedures are being developed to target this pathway, such as small molecules that disrupt the KEAP1-NRF2 interaction or stabilize NRF2 to enhance the antioxidant response [109]. Showing solid affinity for the **KEAP-1** receptor, compound 57 bound through three conventional hydrogen bonds and one alkyl bond, illustrating notable binding energy of −7.7 kcal/mol. In differentiate, Compound 56 connecting through one conventional hydrogen bond and one pi alkyl bond, with a binding energy of −5.6 kcal/mol. By comparison, Ascorbic acid shown a binding energy of −10.6 kcal/mol, including four conventional hydrogen bonds (**Table 6**, **Fig 2**).

**Fig 3:**
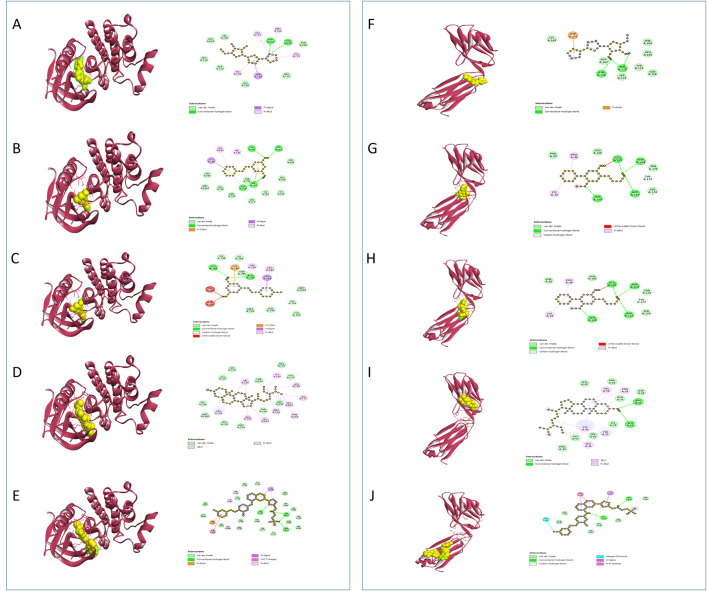
Predicted 3D and 2D interactions of phytocompounds with EGFR and HER2. Panels (A–E) illustrate the binding of C13, C54, C59, C65, and the standard lapatinib with EGFR, respectively. Panels (F–J) illustrate the binding of C13, C57, C61, C65, and the standard lapatinib with HER2, respectively.

**EGFR**, a receptor tyrosine kinase basic for cell growth, differentiation, and survival, belongs to the ERBB receptor family. It activates upon binding to ligands such as epidermal growth factor (EGF) and transforming growth factor-alpha (TGF-α). Activation of EGFR initiates signaling pathways like PI3K-AKT-mTOR and RAS-RAF-MEK-ERK, which regulate genes such as c-Myc (proliferation), Bcl-2 (survival), and Cyclin D1 (differentiation). Dysregulation of EGFR signaling in cancer is often caused by mutations, amplifications, or overexpression, which results in increased cell proliferation, reduced apoptosis, and resistance to therapies like chemotherapy and radiation [110,111]. While **HER2** (Human Epidermal Growth Factor Receptor 2) is a protein that carries out the regulation of cell growth in a similar manner. However, in certain cancers, such as breast cancer, excessive production of HER2 leads to uncontrolled cell growth and proliferation. The development of cancer treatments requires targeting this overexpression [112,113]. Our study revealed that, compound 65 illustrated noteworthy binding affinity to **EGFR**, enlisting −9.6 kcal/mol, slightly lower than Lapatinib’s −10.0 kcal/mol. This interaction included two bonds in Compound 65: five alkyl bonds and five pi alkyl bonds. Essentially, Compound 65 associating with **HER2**, shaping two conventional hydrogen bonds, four alkyl bonds, and one pi alkyl bond, coming about in a binding affinity of −6.9 kcal/mol, associated to Lapatinib as point by point in **Table 6** and **Fig 3**.

**Fig 4:**
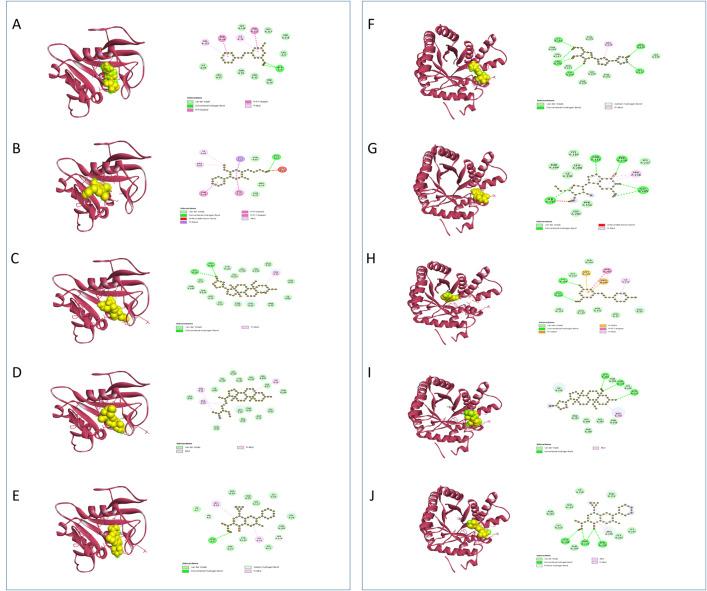
Predicted 3D and 2D interactions of phytocompounds with DHFR and DHPS. Panels (A–E) show the binding of C54, C57, C61, C65, and the standard ciprofloxacin with DHFR, respectively. Panels (F–J) show the binding of C13, C28, C57, C61, and the standard ciprofloxacin with DHPS, respectively.

**DHFR** (Dihydrofolate Reductase) plays a crucial role in cellular metabolism by catalyzing the reduction of dihydrofolate to tetrahydrofolate, a necessary step in the synthesis of purines, thymidylate, and certain amino acids. This process is essential for DNA synthesis and cell replication in both microbial organisms and human cells. In antimicrobial therapy, DHFR is targeted by drugs known as dihydrofolate reductase inhibitors (DHFRIs) which inhibit the enzyme’s activity by disrupting folate metabolism in microbial cells, leading to impaired DNA synthesis and cell death [114,115]. The **Table 6** and **Fig 4** manifested that compound 61 is appeared to connected with the **DHFR** receptor through two conventional hydrogen bonds and one pi alkyl bond, illustrating a noteworthy binding affinity of −8.3 kcal/mol, marginally outperforming the standard Ciprofloxacin with −8.2 kcal/mol, which shapes a conventional hydrogen bond, two carbon hydrogen bonds, and two pi alkyl bonds. Additionally, compound 61 is appeared to connected with the **DHFR** receptor through two conventional hydrogen bonds and one pi alkyl bond, illustrating a noteworthy binding affinity of −8.3 kcal/mol, marginally outperforming the standard Ciprofloxacin with −8.2 kcal/mol, which shapes a conventional hydrogen bond, two carbon hydrogen bonds, and two pi alkyl bonds.

Another receptor, **DHPS** (Dihydropteroate Synthase) is also an enzyme involved in the folate synthesis pathway in microbial organisms. It catalyzes the condensation of p-aminobenzoic acid (PABA) with 6-hydroxymethyl-7,8-dihydropterin pyrophosphate to form dihydropteroate. The production of tetrahydrofolate requires this process, which is essential for nucleotide and amino acid synthesis in bacteria and other microorganisms. DHPS is targeted by sulfonamide antibiotics, which competitively inhibit its activity competitively and sulfonamide disrupt the folate synthesis pathway in bacteria, leading to impaired growth and replication. These findings highlight the importance of **DHPS** as a major target in antimicrobial therapy for bacterial infections [116,117]. In our study, compound 61 moreover appears considerable binding potential of −8 kcal/mol with DHPS, interfacing through three conventional hydrogen bonds and one alkyl group, driving to a binding energy of −7.2 kcal/mol. In differentiate, the reference Ciprofloxacin appears particular interactions with **DHPS**, counting a conventional hydrogen bond, pi donor hydrogen bond, alkyl bond, and pi alkyl bond, yielding a binding energy of −7.2 kcal/mol (**Table 6**, **Fig 4).**

**Fig 5:**
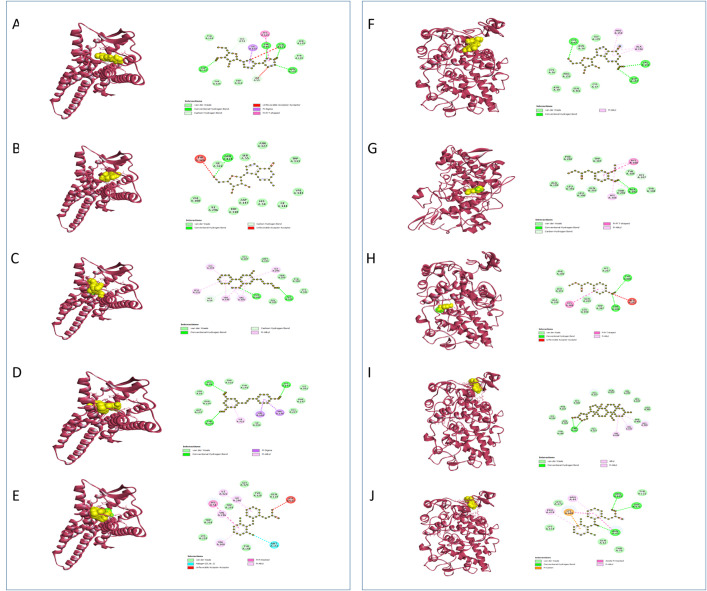
Predicted 3D and 2D interactions of phytocompounds with MOR and COX2. Panels (A–E) represent the binding of C13, C28, C57, C59, and the standard diclofenac with MOR, respectively. Panels (F–J) represent the binding of C28, C40, C56, C61, and the standard diclofenac with COX2, respectively.

Mu-Opioid Receptor (**MOR**) is the primary target of opioid painkillers because it involves the body’s response to pain. It belongs to the family of G protein-coupled receptors (GPCRs) and is transcendently found in the brain, spinal cord, and peripheral nervous system. When activated by endogenous ligands (like endorphins) or exogenous opioids (such as morphine, fentanyl, and oxycodone), **MOR** initiates a series of intracellular events that lead to analgesia, or pain relief. Upon activation, **MOR** inhibits adenylate cyclase activity, reduces cyclic adenosine monophosphate (cAMP) levels, and decreases the release of neurotransmitters such as substance P and glutamate. These actions assist in reducing pain by suppressing the transmission of pain signals and modulating pain perception. Furthermore, MOR activation causes potassium channels to open and calcium channels to close, which leads to hyperpolarization of neurons and further inhibition of neuronal excitability [118,119]. The compound 28 locked in with an amino acid and formed a conventional hydrogen bond along with a carbon hydrogen bond. In contrast, compound 13 experienced two steric clashes but built up four conventional hydrogen bonds, two carbon hydrogen bonds, one pi sigma bond, and one pi pi T shaped bond, resulting in a binding energy of −7.6 kcal/mol, surpassing Diclofenac’s standard binding score of −7.3 kcal/mol. Diclofenac itself employs a halogen bond, an unfavorable donor-donor bond, a pi stacked interaction, and four pi alkyl bonds in its binding mechanism (**Table 6**, **Fig 5**).

**COX-2** (Cyclooxygenase 2) is inducible and typically expressed in response to inflammatory stimuli, such as cytokines and growth factors and it becomes elevated during inflammation.Its activation to increased leads production of prostaglandins, particularly prostaglandin E2 (PGE2), which sensitizes nerve endings and contributes to pain and swelling. In treatment of analgesia nonsteroidal anti-inflammatory drugs (NSAIDs) and selective COX-2 inhibitors (coxibs) target COX-2 to reduce pain and inflammation. By inhibiting **COX-2**, these medications reduce the synthesis of prostaglandins, thereby decreasing inflammation and providing relief from pain [120,121]. Here, Compound 28 surpasses the standard value of Diclofenac, foming a complex with the receptor through the engagement of three conventional hydrogen bond and two pi alkyl bond (**Table 6**).

**Fig 6:**
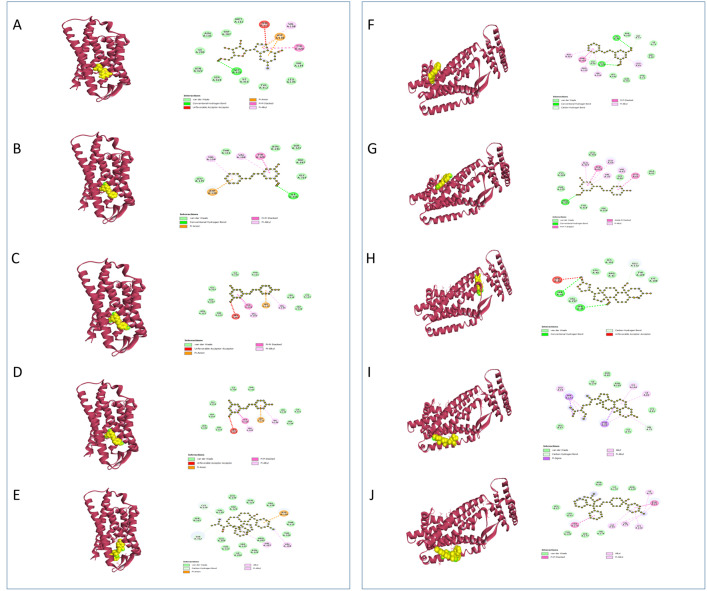
Predicted 3D and 2D interactions of phytocompounds with KOR and δOR. Panels (A–E) show the binding of C28, C54, C59, C61, and the standard loperamide with KOR, respectively. Panels (F–J) show the binding of C54, C59, C61, C65, and the standard loperamide with δOR, respectively.

Kappa Opioid Receptors (**KOR**) belong to the G protein-coupled receptor family and are found widely throughout both the central and peripheral nervous systems Regulating pain perception, mood, and various gastrointestinal functions is an essential role played by them. Specifically, within the gastrointestinal tract, activating KORs reduces gastrointestinal motility and decreases fluid secretion into the intestinal lumen. This leads to slower movement of food through the intestines and fewer bowel movements, which can be potentially alleviating symptoms of diarrhea [122]. Similarly, in gastrointestinal function, **δOR** (delta opioid receptors) are involved in regulating intestinal motility and fluid secretion. Activation of **δOR** in the gastrointestinal tract decreases intestinal motility and reduces fluid secretion into the intestinal lumen. These actions help slow intestinal transit and reduce the frequency of bowel movements, suggesting that targeting **δOR** activation could be beneficial for developing treatments to alleviate diarrhea [123]. Our investigation reveals that several compounds listed in Table 4 have significant binding affinity for both **KOR** and **δOR**. Specifically, compounds 54 and 59 showed strong binding with these receptors, achieving binding affinity scores of −7.9 kcal/mol and −8 kcal/mol, respectively, for KOR. In comparison, the standard Loperamide interacted with four amino acids through two conventional hydrogen bonds and three other bond types (**Table 6**, **Fig 6**). Moreover, compounds 54 and 59 formed interactions with eight and seven amino acids for δOR, resulting in binding values of −8.4 kcal/mol and −7.6 kcal/mol, respectively. Conversely, the reference Loperamide exhibited a binding interaction value of −8.7 kcal/mol, involving two pi pi stacked bonds, three pi alkyl bonds, and three alkyl bonds.

The ADME/T (Absorption, Distribution, Metabolism, Excretion, and Toxicity) analysis of the highest binding compounds revealed significant variations in their pharmacokinetic and safety profiles, which are crucial for evaluating their potential as drug candidates. Among the tested compounds, C54, C57, C59, and C61 demonstrated particularly favorable properties, including high gastrointestinal absorption, no predicted AMES toxicity or hepatotoxicity, and good bioavailability scores of 0.55, along with compliance to drug-likeness criteria (**Table 7**). These compounds exhibited moderate lipophilicity (log P values between 1.33 and 3.02) and an optimal balance of hydrogen bond donors and acceptors, which are known to influence membrane permeability and solubility [20,124]. The high GI absorption predicted for these compounds suggests their potential for oral bioavailability, an important consideration for therapeutic development [125]. In contrast, compounds C13 and C28 showed limitations such as low GI absorption and violations of drug-likeness rules, primarily due to their high topological polar surface area (TPSA > 150 Å²) and extremely low log P values (−1.98 and −1.94, respectively), which typically correlate with poor membrane permeability [126]. Interestingly, C65, despite showing strong binding affinity in molecular docking studies, displayed extremely high lipophilicity (log P 7.8) and low GI absorption, likely due to poor water solubility, which could limit its practical utility despite its promising target engagement [127]. The absence of AMES toxicity predictions across all compounds indicates a low risk of mutagenicity, while the hepatotoxicity observed only in C13 and C28 warrants caution for these particular molecules [128]. The bioavailability scores, consistently at 0.55 for most compounds except C13 (0.11), suggest generally favorable pharmacokinetic profiles, particularly for C54, C57, C59 and C61 which combined these attributes with drug-like properties. These ADME/T results complement the earlier binding affinity data, providing a more comprehensive picture of the compounds’ therapeutic potential, where good target binding must be balanced with suitable pharmacokinetic properties for successful drug development [129]. The findings underscore the importance of such computational ADME/T screening in early drug discovery to identify lead compounds with optimal balance between potency and drug-like characteristics, potentially saving considerable time and resources in subsequent preclinical development [130]. Recent studies have also emphasized the value of integrating molecular docking with ADME/T predictions to streamline phytochemical-based drug discovery, further validating the approach adopted in this work [131–133]

**Table 7:** ADME/T analysis of highest binding compounds towards the target receptors.

Compound No	H-bond Donor	H-bond Acceptors	Lipophilicity – log P (o/w)	GI Absorption	AMES Toxicity	Hepatotoxicity	Bioavailability score	Drug likeliness
**C13**	4	9	−1.98	Low	No	Yes	0.11	No; 2 violations: XLOGP3 < −2, TPSA>150
**C28**	5	7	−1.94	Low	No	Yes	0.55	No; 1 violation: TPSA>150
**C54**	2	2	2.90	High	No	No	0.55	Yes
**C56**	1	3	1.43	High	No	No	0.55	No; 1 violation: MW < 200
**C57**	2	3	3.02	High	No	No	0.55	Yes
**C59**	3	3	2.48	High	No	No	0.55	Yes
**C61**	4	6	1.33	High	No	No	0.55	Yes
**C65**	1	1	7.8	Low	No	No	0.55	No; 1 violation

### Limitations

This study delivers a comprehensive GC-MS/MS profile, bioactivity evaluation, molecular docking, and ADME/T predictions for *M. oleifera* methanolic leaf extract; however, certain aspects remain to be explored further. While the docking scores and *in silico* ADME/T results offer valuable preliminary insights, additional *in vitro* and *in vivo* validation will help confirm the pharmacological potential of the identified compounds. Some constituents were present at relatively low concentrations, suggesting that future enrichment or purification could enhance their biological relevance. The antimicrobial, antidiarrheal, and analgesic assays were conducted under defined experimental conditions, and expanding these into broader models may better reflect real-world applications. For a few compounds with strong binding affinity but less favorable predicted pharmacokinetics, structural refinement or advanced formulation approaches could improve their drug-likeness. Finally, as toxicity evaluation in this work was limited to computational predictions, complementary experimental safety studies would be a logical next step before clinical translation.

## Conclusion

This study comprehensively evaluated *Moringa oleifera* through biological assays and computational approaches, identifying compounds with strong target engagement and favorable pharmacokinetic profiles. GC-MS/MS characterization revealed numerous bioactive compounds, including phytosterols, phenolic compounds, and terpenes, which align with the plant’s traditional medicinal uses. The extract showed measurable antibacterial activity against both Gram-positive (16–17 mm inhibition zones for *S. aureus* and *B. cereus*) and Gram-negative strains (19 mm for *P. aeruginosa* and *S. typhi*), albeit weaker than synthetic antibiotics (26–42 mm). In antidiarrheal testing, the extract (400 mg/kg) reduced castor oil-induced diarrheal episodes by 58.06%, while in analgesic evaluation, it decreased acetic acid-induced writhing by 59.18% at the same dose. Molecular docking highlighted strong binding affinities of key compounds [56,60,64] against critical targets like URO, KEAP-1, EGFR, and DHFR, suggesting therapeutic potential in oxidative stress, inflammation, and microbial infections. ADME/T analysis further distinguished truly drug-like candidates (C57, C59, C61) from less favorable ones (C13, C28). The lead compound C57 was particularly noteworthy, combining strong binding affinity with optimal physicochemical and safety profiles. Overall, *M. oleifera* emerges as a promising source of multi-target therapeutic agents, with future work focusing on compound isolation, in vivo validation, and exploration of synergistic effects to enhance bioactivity.

## Supporting information

S1 File Meta data of *In vitro* and *In vivo* experiments. (XLSX)

S1 Fig Zone of inhibition assay showing five replicate discs of the methanolic extract of *Moringa oleifera* (100 μg/disc) placed around a central standard antibiotic disc (30 μg/disc) against a single bacterial strain. Clear inhibition zones demonstrate the relative activity of the plant extract compared to the standard.
(TIF)
