# Supplementary figures and images for: Phytochemical profiling and bioactivity validation of Moringa oleifera leaves: Antimicrobial, antidiarrheal, analgesic, and in silico insights
Source: PLoS One. 2025 Sep 12;20(9):e0332048. doi: 10.1371/journal.pone.0332048 (PMC12431276; doi:10.1371/journal.pone.0332048)

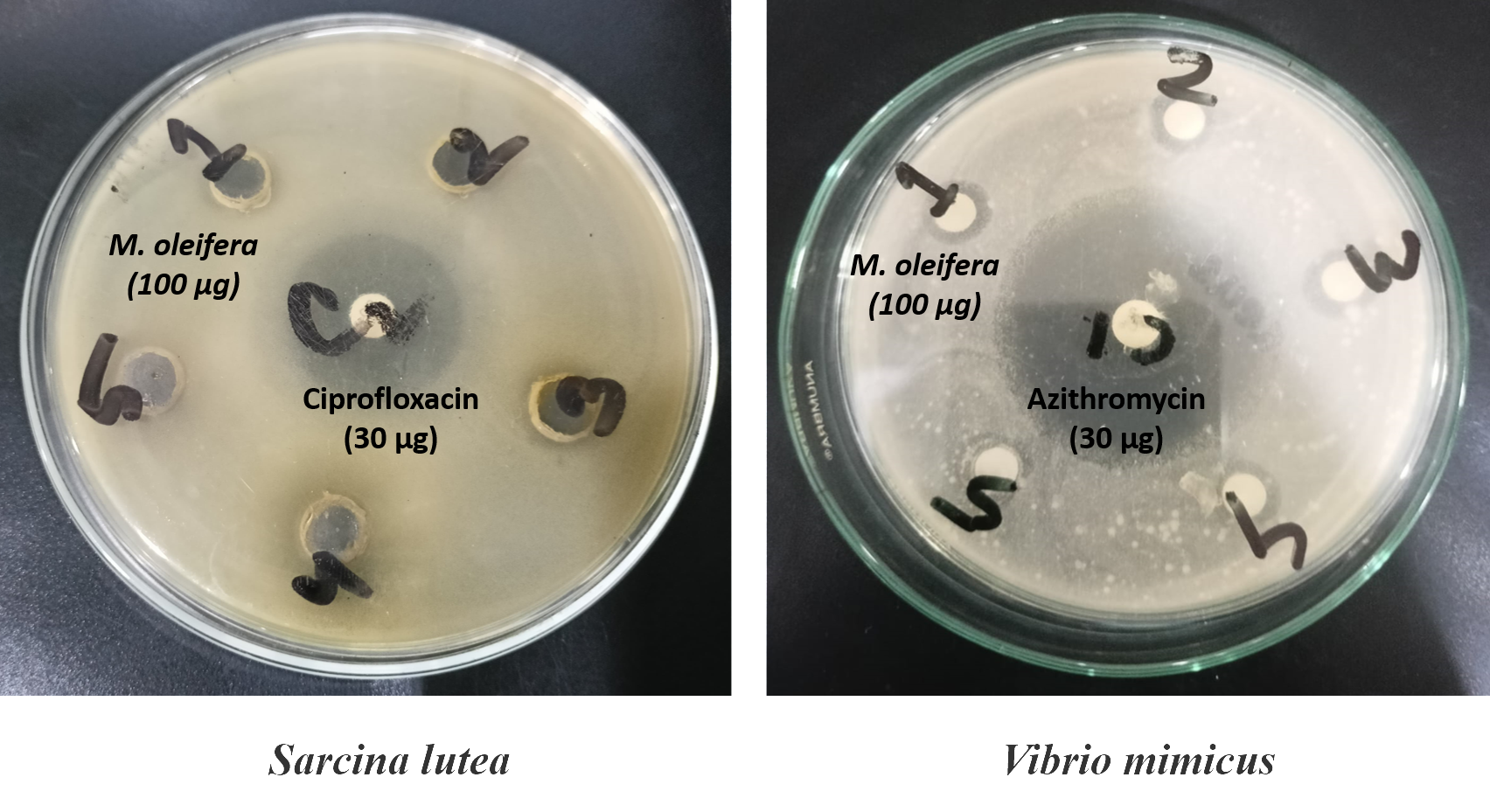

Supplement: S1 Fig — Clear inhibition zones demonstrate the relative activity of the plant extract compared to the standard. (TIF) [file pone.0332048.s002.tif]
